# Granger Causality Network Reconstruction of Conductance-Based Integrate-and-Fire Neuronal Systems

**DOI:** 10.1371/journal.pone.0087636

**Published:** 2014-02-19

**Authors:** Douglas Zhou, Yanyang Xiao, Yaoyu Zhang, Zhiqin Xu, David Cai

**Affiliations:** 1 Department of Mathematics, MOE-LSC, and Institute of Natural Sciences, Shanghai Jiao Tong University, Shanghai, China; 2 Courant Institute of Mathematical Sciences and Center for Neural Science, New York University, New York, New York, United States of America; 3 NYUAD Institute, New York University Abu Dhabi, Abu Dhabi, United Arab Emirates; Universiteit Gent, Belgium

## Abstract

Reconstruction of anatomical connectivity from measured dynamical activities of coupled neurons is one of the fundamental issues in the understanding of structure-function relationship of neuronal circuitry. Many approaches have been developed to address this issue based on either electrical or metabolic data observed in experiment. The Granger causality (GC) analysis remains one of the major approaches to explore the dynamical causal connectivity among individual neurons or neuronal populations. However, it is yet to be clarified how such causal connectivity, i.e., the GC connectivity, can be mapped to the underlying anatomical connectivity in neuronal networks. We perform the GC analysis on the conductance-based integrate-and-fire (I
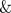
F) neuronal networks to obtain their causal connectivity. Through numerical experiments, we find that the underlying synaptic connectivity amongst individual neurons or subnetworks, can be successfully reconstructed by the GC connectivity constructed from voltage time series. Furthermore, this reconstruction is insensitive to dynamical regimes and can be achieved without perturbing systems and prior knowledge of neuronal model parameters. Surprisingly, the synaptic connectivity can even be reconstructed by merely knowing the raster of systems, i.e., spike timing of neurons. Using spike-triggered correlation techniques, we establish a direct mapping between the causal connectivity and the synaptic connectivity for the conductance-based I
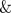
F neuronal networks, and show the GC is quadratically related to the coupling strength. The theoretical approach we develop here may provide a framework for examining the validity of the GC analysis in other settings.

## Introduction

The relation between structure and function is one of the central research themes in biology. In order to fully understand the function of biological organisms, it is often important to analyze the structure of the systems [1]–[3]. The characterization of structure can be different with respect to the scales one is interested in. On the molecular level, the structure may refer to microscopic configurations of atoms, e.g., in hierarchical protein folding. Whereas, at the system level, such as neuronal circuitry, the structure often refers to the anatomical connections amongst neurons. To find the wiring diagram, i.e., synaptic connectivity, is often regarded as a key step towards understanding of the information processing and function of the brain [4]–[6]. New experimental observation tools, such as diffusion tensor imaging, are useful to tract fiber pathways in the whole brain, however, they usually have an insufficient spatial resolution and cannot be used to infer connections at the cellular level. Systematic assessment of global network synaptic connectivity through direct electrophysiological assays has remained technically infeasible, even for some simple systems such as dissociated neuronal culture [7]–[9]. However, it is relatively easy in experiment to obtain dynamical activities of neuronal populations or individual neurons through, e.g., local field potential, spike trains measurement, magnetoencephalography (MEG), electroencepholography (EEG), or functional magnetic resonance imaging (fMRI). Based on experimentally measured data, many network analysis approaches have been developed in attempt to probe the underlying brain connectivity through various statistical approaches [10]–[13], such as Granger causality [14]–[16] and dynamic Bayesian inference [17], [18]. Through these analyses, the obtained connectivity is often referred to as functional or effective connectivity [19]. However, such functional (effective) connectivity obtained from different computational analysis is often different from one another [20], [21]. Conceptually, they are also different from the structural (synaptic) connectivity. To infer the underlying network structure from observation, it is desirable to explore the relationship between structural and functional connectivity [22]–[25]. Understanding of how the functional connectivity is mapped to the anatomical synaptic connectivity in the brain remains one of the major challenges in systems neuroscience [26]–[29].

In this work, we study the relationship between structural connectivity and a particular functional connectivity which we will describe presently for conductance-based integrate-and-fire (I
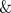
F) neuronal networks. It has been shown in experiment that I
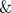
F models can statistically faithfully capture the response of cortical neurons under in-vivo-like currents in terms of both firing dynamics and subthreshold membrane dynamics [30]–[33]. In theoretical and computational neuroscience, the conductance-based I
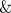
F neuron has served as an efficient reduced model of cortical neurons to study their statistical spike-encoding properties [34], [35]. For instance, the I
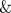
F neuron has been widely used as basic neuronal units for modeling large-scale cortical dynamics to investigate information processing in certain areas of the brain [36]–[42]. In our study, the structural connectivity of I
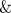
F networks denotes synaptic connections between neurons, which are characterized by the adjacency matrix of the network. The particular functional connectivity of I
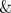
F networks in our work denotes the connectivity constructed by the Granger causality (GC) analysis. The notion of GC was originally introduced by Wiener to determine causal influence from one dynamical variable 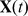 to the other 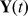 [43]. It was further mathematically formulated using linear regression/prediction models [43]–[45]. In this framework, if the prediction of 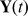 can be improved by incorporating the information in the history of 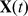, it is said that there exists a causal connection from the time series 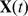 to 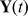. Due to its simplicity and easy implementation, the GC theory has been extensively applied to study the functional connectivity of networks in neuroscience as well as in other scientific fields such as systems biology, medical engineering, economics, and social science [14], [46]. By using voltage or spike train time series obtained from the I
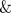
F network dynamics, the functional connectivity of I
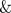
F networks can be obtained from the GC analysis, which we will term as the GC connectivity, and describe this connectivity by the causal adjacency matrix.

The main theoretical issue we address in this work is whether we can establish a direct, quantitative mapping between the structural connectivity and the GC connectivity for I
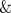
F neuronal networks. That is, whether the underlying structural connectivity, which is usually not easy to assess in experiment, can be extracted by using the GC analysis. There are several challenges in this task: (i) the GC theory is based on linear regression models and assumes that the causal relationship can be well captured by low order statistics (up to the variance) of signals, e.g., Gaussian time series [47]. Theoretically, it has yet to determine whether the linear GC framework is applicable to I
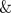
F systems, whose dynamics are nonlinear and non-smooth; (ii) the notion of GC connectivity is statistical rather than structural, i.e., quantification of directed statistical correlation between dynamical elements, whereas the structural connectivity corresponds to physical connections between dynamical units. A priori, there is no obvious reason that these two types of connectivity are always identical to each other [9], [21], [48]. For instance, there were indications that strong effective connections could exist between regions with no direct structural connections [23], [49], [50] and the functional connectivity could vary under different dynamical states associated with the same structural network [3], [51].

We first develop a reliable numerical algorithm for obtaining the GC connectivity of I
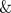
F networks. Through numerical studies, we show that the GC connectivity is highly coincident with the structural connectivity, i.e., the synaptic connectivity between neurons in a network can be well reconstructed by the causal connectivity obtained from the GC analysis on voltage time series. We point out that this reconstruction can be achieved despite the fact that the dynamics of I
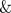
F networks are both nonlinear and non-smooth. As demonstrated in our numerical results, this reconstruction is quite robust as long as the time series are reasonably long for the system to reach a statistically steady state. The reconstruction is also insensitive to the system size and is independent of dynamical regimes. We then investigate the theoretical underpinning of this network reconstruction by means of the spike-triggered correlation (STC) approach. Our analysis shows that the STC on voltage time series, often a standard method used for inference of connectivity in experiment [52], [53], cannot capture the correct inference of the underlying synaptic connections between neurons. This failure has to do with the fact that voltage signals usually have a finite autocorrelation time. We further show that the STC on voltage-signal residuals, i.e., whitened signals obtained from regression models, is able to link the GC connectivity and the structural connectivity of the network. This is achieved by first establishing the structure of STC on residuals to reflect the underlying coupling between neurons, then showing this STC is linearly related to residual cross-correlations. Further, by solving the Yule-Walker equations with respect to residuals, we can obtain a relation between GC and the residual cross-correlations for the I
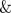
F networks, thus connecting GC to the underlying coupling between neurons through STC on residuals. In addition, we can obtain the relationship that GC for neuron 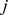 to neuron 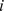 is proportional to 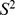, where 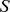 is the synaptic coupling strength from neuron 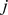 to neuron 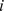.

To investigate the range of applicability of our method, we further demonstrate that the GC analysis is also capable of detecting synaptic connections between individual neurons and a subnetwork of neurons (i.e., a group of interacting neurons), or connections between subnetworks. This is motivated by the signals measured by extracellular recordings in experiment, i.e., the local field potential. Our results indicate that the synaptic connection may also be detected from measured signals between intracellular (individual neuron) and extracellular recordings (a group of neurons, i.e., subnetworks). In addition, we show that the network reconstruction through the GC theory can also be achieved using spike train time series. In comparison with the precise voltage-trace measurement, we note that spike train time series are relatively easy to measure in experiment, thus, rendering spike-train GC analysis particularly useful for practical settings. This is rather striking in that one can essentially reconstruct the synaptic connectivity of I
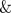
F networks by only examining the raster plot of a group of neurons. In addition, we also demonstrate that our reconstruction can be extended to networks with both excitatory and inhibitory neurons, or to more realistic neuronal networks, e.g., of the exponential I
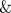
F neurons. Note that our results provide a direct link between the GC connectivity and the structural connectivity with no intervention of systems and no prior knowledge of neuronal model parameters. Therefore, this method may be potentially useful in experiment to infer the structural information of neuronal networks. Because the GC theory is often used to investigate the direction of information flow within networks, our work may also shed light on how propagation of information flow within networks can be influenced by the network topology.

## Results

The systems we study are conductance-based, integrate-and-fire type neuronal networks [See Eqs. (23), (24) and (25) in *Methods*]. As mentioned previously, with in-vivo-like current injection, the I
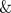
F neuronal model can capture well both the firing rate and subthreshold dynamics of cortical neurons [30], [31]. Consequently, networks of I
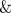
F neurons have served as prototypical theoretical models to provide insight into fascinating dynamics of many neuronal networks in the brain [32], [33], [35], [54].

The Granger causality characterizes causal interactions between time series by distinguishing the driver from the recipient (See theoretical definitions in *Methods*), namely, the driver, which is earlier than the recipient, contains information about the future of the recipient, and thus the variance of the prediction error is reduced when the information of the driver is incorporated. In general, the causal influence between time series reflects a drive-response scenario and this influence can be either reciprocal or unidirectional. As discussed later, such causality which is based on temporality is characterized by the directional correlation relations between time series.

We apply the Granger causality analysis to these widely used I
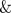
F neuronal networks to investigate the relationship between causal and structural connectivities (See GC algorithm in *Methods*). By applying the GC algorithm to the I
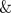
F networks, we can obtain all the GC values from neuron 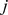 to neuron 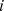, denoted by 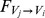, for 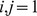, 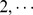,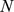. Then, we perform the *p*-value test (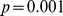 in our simulations) to determine a GC threshold 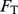 (See Text S1 for more details). If 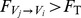, we define that there is a significant causal interaction from the 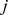
th neuron to the 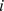
th neuron and denote this by 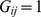. Otherwise, we say there is no causal influence from the 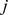
th neuron to the 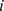
th neuron and denote this by 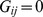. Because GC interactions between two neurons are in general not symmetric, by representing them as edges in a graph, we can define a directed graph or a causal connectivity network, as characterized by the matrix 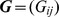, for the I
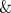
F systems [55], [56]. Meanwhile, the structural connectivity of our I
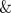
F system is characterized by the synaptic adjacency matrix, denoted by 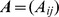 (See *Methods*). Note that, the causal connectivity can be viewed as a type of functional connectivity [19], [29], whereas the structural connectivity reflects physical connectivity. As discussed in the *Introduction*, our causal connectivity is a statistical measure, and it is, in general, not equivalent to the underlying physical connections between dynamical variables [56].

### Causal connectivity vs. structural connectivity for I&F networks

As described above, the GC connectivity can be characterized by the causal adjacency matrix 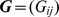, whereas the structural connectivity is characterized by the synaptic adjacency matrix 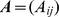, 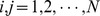. In the following, we discuss the relationship between 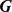 and 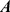 for the I
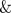
F networks, i.e., the relationship between GC connectivity and structural connectivity.

**Figure 1:**
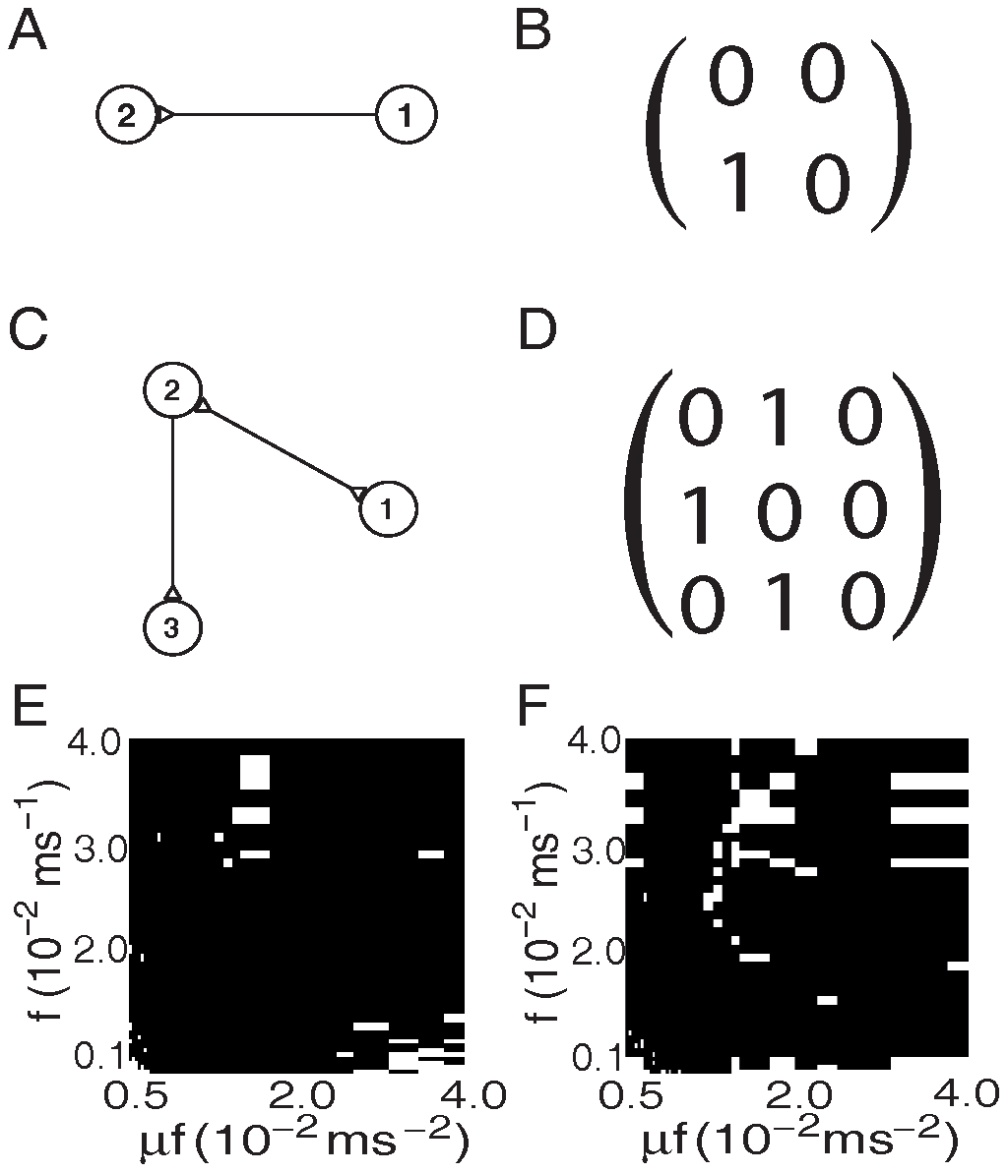
GC connectivity for small excitatory networks. For networks of two excitatory neurons and three excitatory neurons in (A) and (C), the edge with only a triangle at the end signifies a directed connectivity. Parameters in (A)-(D) are chosen as 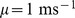 (Poisson input rate), 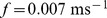 (Poisson input strength), and the coupling strength 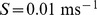 (the corresponding EPSP is 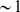
mV). (A) A two-neuron network with only a synaptic connection from neuron 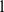 to neuron 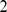. (B) Causal adjacency matrix 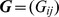 constructed by GC, which captures the synaptic connectivity in (A). (C) A three-neuron network with a synaptic connection from neuron 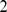 to neuron 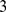 and with bidirectional synaptic connections between neuron 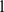 and neuron 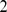. (D) Causal adjacency matrix 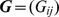 constructed by GC, which captures the synaptic connectivity in (C). The coincidence between the synaptic adjacency matrix 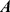 and the causal adjacency matrix 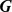 as a function of rate 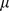 and magnitude 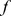 in the Poisson drive for (E) the two-neuron network as shown in (A), and (F) the three-neuron network as shown in (C). The parameter region labeled by the white color indicates that 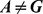, and by the black color indicating that 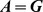.

Figure 1A and C shows examples of synaptic connectivity 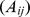 between neurons for a two-neuron and a three-neuron networks. Figure 1B and D displays the corresponding causal adjacency matrix 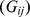 constructed by using our GC algorithm on the voltage time series. It can be clearly seen that the causal connectivity is coincident with the synaptic connectivity. These examples present compelling evidence that the synaptic adjacency matrix of the I
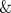
F networks can be successfully reconstructed by using the GC algorithm on neurons' voltage trajectories.

**Figure 2:**
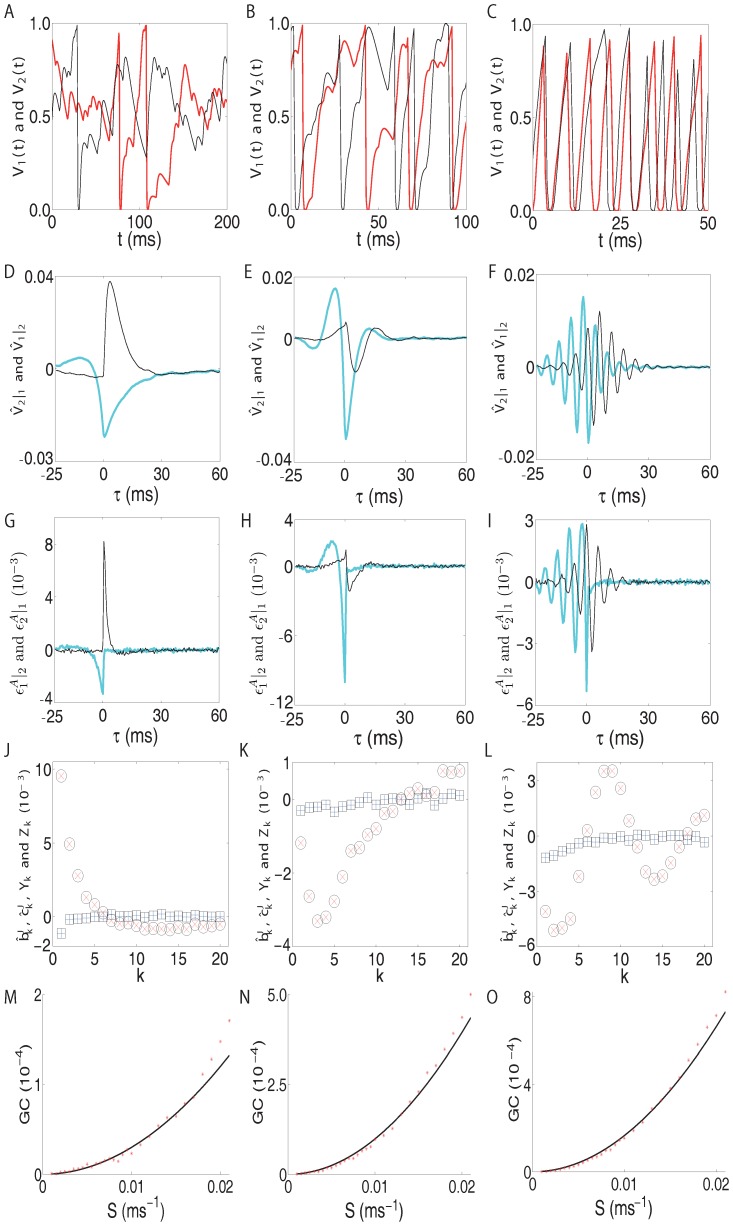
Characteristics of different dynamical regimes. Illustrated here are the dynamic characteristics of the two-excitatory-neuron network in Fig. 1A with different Poisson input rate 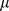 for highly fluctuating regime [(A),(D),(G),(J) and (M)] with 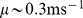, intermediate regime [(B),(E),(H),(K) and (N)] with 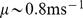 and mean-driven regime [(C),(F),(I),(L) and (O)] with 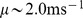. The fixed input strength 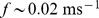. For these three dynamical regimes, we plot the corresponding quantities: (A), (B), and (C) are voltage trajectories 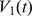 (black online) and 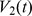 (red online). (D), (E), and (F) are spike-triggered correlation on voltage [Eq. (1)]: 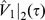 (cyan online) and 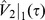 (black online). (G), (H), and (I) are spike-triggered correlation on residuals [Eq. (2)]: 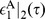 (cyan online) and 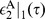 (black online). (J), (K), and (L) are numerically computed regression coefficients 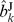 (blue “plus” online), 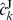 (red “cross” online) and their corresponding approximations 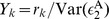 (“square” symbol), 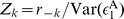 (“circle” symbol). (M), (N), and (O) are the GC 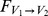 (red “star” online) as a function of coupling strength 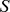, the line (black online) is a quadratic fit.

Next, we address the question of whether these successful reconstructions are merely accidental cases or whether there is a large class of networks that are amenable to this analysis. To examine whether the reconstruction is dependent on particular dynamical regimes, which are often described by a particular choice of network system parameters, we investigate the robustness of the reconstruction by scanning the magnitude 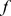 and the rate 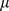 in the Poisson drive of the I
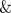
F networks [See Eq. (23) in *Methods*]. The choice of these parameters covers the realistic firing rates (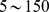
Hz) of real neurons [35], [57]. Note that there are typically three dynamical regimes for the I
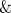
F neurons for each fixed input strength 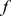: (i) a highly fluctuating regime when the input rate 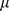 is low; (ii) an intermediate regime when 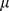 is moderately high; (iii) a low fluctuating or mean driven regime when 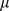 is very high [58], [59]. Figure 2A–C shows the voltage trajectories of two neurons for different choices of input rate 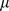 with the input strength 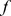 fixed. It can be seen from Fig. 2A–C that the firing pattern is rather irregular when 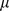 is low (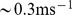), whereas the spiking activity of neurons becomes relatively regular (nearly periodic) when 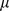 is very high (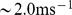). For all these dynamical regimes, we can demonstrate that there is a wide range of the network parameters whose synaptic connectivity can be analyzed using the GC analysis. As shown in Fig. 1E and F, the GC connectivity (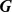) and the synaptic connectivity 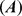 are highly coincident with each other for both two-neuron and three-neuron networks over a wide range of dynamical regimes.

**Figure 3:**
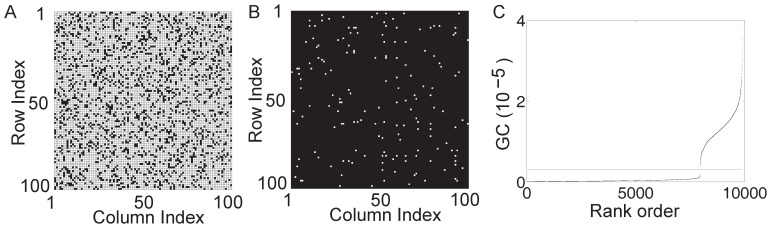
GC connectivity for large excitatory networks. For an I
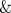
F network of 100 excitatory neurons with random connectivity, the synaptic adjacency matrix 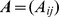 is shown in (A) with the white color indicating that 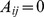 and the black color for 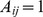. The total number of nonzero 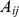 is 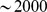 (the percentage of connections is 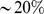) and the average neuronal firing rate is 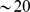
Hz. (B) The absolute difference between 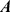 and the causal adjacency matrix 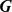, i.e., 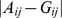. The white color indicates that 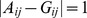, i.e., 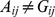 and the black color when 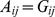. By significance test (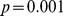, See Text S1 for more details), the total number of 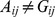 is 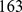 out of 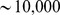 possible pairs of connections. (C) Ranked GC in order of magnitude with the horizontal line (blue online) indicating a threshold in the gap of the ranked GC. Parameters are chosen as 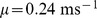 (Poisson input rate), 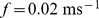 (Poisson input strength), and the coupling strength 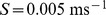 (the corresponding EPSP is 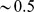
mV).

We further examine whether the synaptic connectivity of large networks with multiple neurons can be revealed by the GC connectivity analysis. For a network of 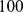 neurons with random connectivity, its synaptic adjacency matrix (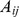) is shown in Fig. 3A, where the total number of nonzero 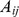, as indicated by the black color, is approximately 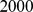. Applying the GC analysis to this network, we can construct its causal connectivity matrix 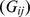. Figure 3B shows the difference between 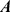 and 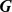, where the white color represents 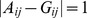, i.e., 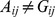, and the black color represents 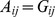. It can be seen that the synaptic adjacency matrix 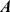 can be successfully reconstructed by the causal adjacency matrix 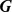 with very high accuracy (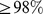). Incidentally, we also point out an interesting phenomenon as observed for the GC connectivity of large excitatory neuronal networks: if we rank the GC by magnitude for all possible directed connections between neurons, there often is a gap separating these ranked GC values as indicated by the gray horizontal line (blue online) in Fig. 3C. This gap clearly divides the GC values into two distinct groups. Surprisingly, by using this gap, for example, by choosing a horizontal line within the gap as the GC threshold 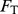, we obtain that 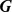 is identical to 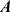.

### Mechanism underlying the successful reconstruction

In this section, we address the issue of why the GC framework, based on linear systems, can be used to reveal the synaptic connectivity of nonlinear network dynamics of I
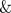
F neurons. For dynamical systems of pulse-coupled type, such as I
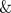
F neurons, the spike-triggered correlation (STC) or spike-triggered averaging method has been widely applied in studies of synaptic connections in such systems [52], [60]. The STC on voltages from the 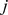
th neuron to the 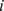
th neuron is defined as
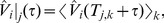
(1)where 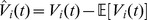 has zero mean, 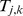 is the 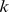
th spike time of the 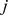
th neuron as defined in Eq. (23) (See *Methods*) and 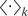 is the average with respect to 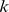, i.e., average over all spikes of the 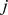
th neuron. Note that, the STC contains the information of both the statistics of the spike drive from the 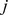
th neuron and the response of the 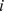
th neuron [52], [60]. Therefore, this drive-response scenario apparently reflects the causal connectivity from the 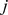
th neuron to the 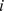
th neuron. On the other hand, the existence of this drive-response relation might imply the existence of synaptic connectivity from the 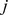
th neuron to the 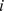
th neuron, i.e., 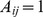. Therefore, it appears that the feature of STC on voltage can be used to relate the causal connectivity to the synaptic connectivity for the I
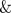
F network system.

For the two-neuron network in Fig. 1A, the STCs on voltages between neuron 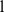 and neuron 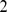 [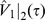 and 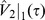] in the three different dynamical regimes are displayed in Fig. 2D–F. From the definition of STC [Eq. (1)], if the 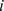
th neuron's response 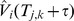, averaged over all the spikes of the 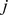
th neuron, exhibits significant deviations from zero when 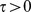, it might imply that the 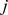
th neuron is presynaptic to the 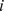
th neuron, otherwise 
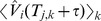
 should be nearly zero after statistical average [52], [60], [61]. However, as shown in Fig. 2D–F, both STCs, 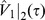 and 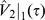, exhibit significant deviations from zero for 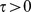 when 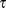 is small and naturally vanish when 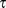 is sufficiently large in all dynamical regimes shown in Fig. 2A–C. These nonzero features in STCs, 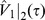 and 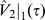, may suggest that the connections between two neurons are bidirectional [61], [62]. However, from the network synaptic connectivity as shown in Fig. 1A, there is only a unidirectional synaptic connection from neuron 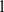 to neuron 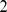. Therefore, one needs to address the question of why the STC 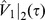, similarly 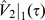, exhibit nonzero features for 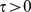 despite the fact that there is no synaptic connection from neuron 2 to neuron 1. Intuitively, we can understand the phenomenon as follows: because the voltage signal 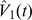 is not white, i.e., there is a finite correlation time for the voltage signal, the future of 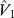 will be correlated with its own history. On the other hand, neuron 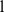 is presynaptic to the neuron 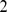, thus giving rise to the possibility that 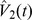 is also correlated with the history of 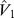. Therefore, 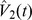 would be likely correlated with the future of 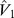. This correlation is reflected in the nonzero feature of the STC 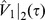 for 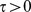, and it can give rise to an incorrect inference of the synaptic connection from neuron 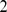 to neuron 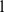.

From the above argument, the nonzero feature of the STC 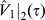 is closely related to the finite-time autocorrelation structure of voltage signals. This has led us to investigate the STC on signals without finite-time autocorrelations, i.e., whitened signals, in order to extract correct synaptic connectivity between neurons. Note that, the residuals 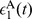 and 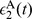, as obtained in auto regression (AR) models [See Eq. (17) in *Methods*], are whitened signals [44], [45], i.e., with only instantaneous correlation. Therefore, we may study the STC on residuals 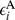 and 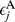:
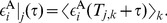
(2)As shown in Fig. 2G–I, for 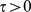, the STC 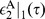 possesses similar features to that of the STC 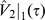, indicating the existence of synaptic connectivity from neuron 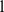 to neuron 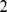. However, unlike the STC 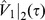, the STC 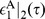 statistically vanishes for 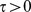, suggesting that neuron 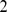 is not presynaptic to neuron 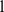. These results indicate that the STC on residuals, i.e., whitened signals, can provide a correct inference about the unidirectional connection between the two neurons.

As the STC on residuals may be used to successfully detect the synaptic connectivity between neurons, it is natural to ask whether the GC connectivity between the signals of residuals are related to the underlying mechanism for the success of the reconstruction of networks. From the AR models for 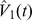 and 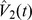 [See Eq. (17) in *Methods*], we can construct the moving average representations of 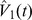, 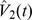 in terms of residuals 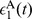, 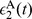 [63], [64].
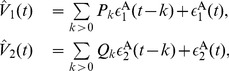
(3)where 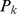 and 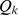 are constant coefficients. Then, substituting Eq. (3) into the joint regression (JR) models for 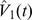 and 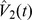 [See Eq. (18) in *Methods*], we obtain the corresponding JR models for 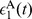 and 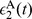:
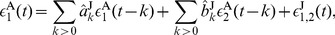
(4a)

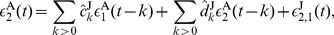
(4b)where 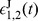 and 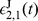 are the same residuals as those in the original JR models for 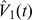 and 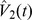 [See Eq. (18) in *Methods*]. Note that Eqs. (4a) and (4b) can also be obtained by using the least-squares method. On the other hand, we can construct the AR models for 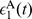 and 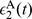 as
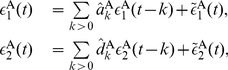
(5)Eqs. (4) and (5) represent JR and AR processes for residuals 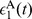 and 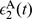, respectively. By the definition of GC, we can obtain 
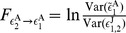
 and 
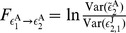
. Note that the residuals 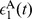 and 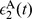 are whitened signals. Therefore, the coefficients 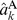, 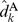 in the AR models (5) are zero and we have 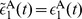, 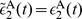. This yields that the GC is invariant as
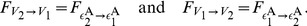
(6)From Eq. (6), it can be seen that the causal connectivity is indeed embedded in the whitened residuals 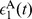 and 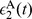. In the following, we will show how the STC on residuals bridges the causal connectivity and the synaptic connectivity.

We first derive analytical expressions of GC for the I
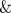
F networks and show that they are closely related to the residual cross-correlation between 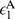 and 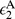. Multiplying Eq. (4a) by the residual 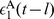 or 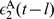, for 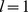, 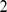, 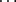, and taking expectations, we obtain the Yule-Walker equations [63], [64] with respect to the coefficients 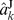 and 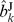 as
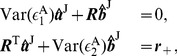
(7)where 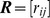 is the covariance matrix with 
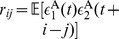
. The column vectors 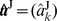, 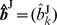 and 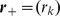, for 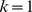, 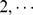, where 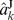, 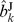 and 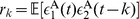 are the 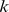
th component in the vectors. Similarly, if we multiply Eq. (4b) by the residual 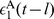 or 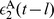, for 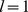, 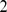, 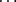, and take expectations, then we can obtain the Yule-Walker equations with respect to coefficients 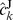 and 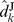


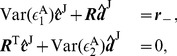
(8)where the column vectors 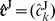, 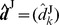 and 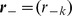, for 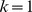, 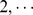, where 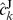, 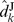 and 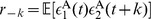 are the 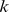
th component in the vectors. Solving Eqs. (7) and (8), we obtain the regression coefficients as
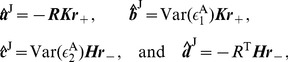
(9)where the matrices 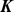 and 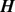 are defined as 
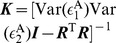
 and 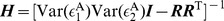 with 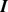 being the identity matrix.

As mentioned previously, Eqs. (4) can also be obtained by using the least-squares method. From this viewpoint, by multiplying Eq. (4a) by 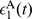 and Eq. (5) by 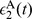, then taking expectations, we can obtain
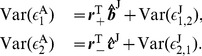
(10)Substituting Eqs. (9) into Eqs. (10) and using the GC definition [See Eqs. (19) in *Methods*], we arrive at
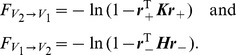
(11)For small residual cross-correlation between 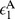 and 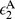, which is consistent with our numerical simulation results for the I
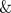
F networks, namely, 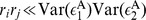 with 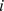, 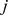 chosen as any integers, the matrices 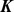 and 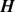 can both be approximated by 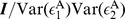. Therefore, the regression coefficients 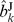 and 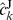 in Eqs. (9) can be approximated by
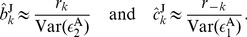
(12)As verified numerically in Fig. 2J–L, Eqs. (12) provide very good approximations of the regression coefficients 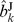 and 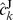 for the I
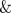
F networks. We observe that 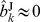 and 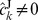 as shown in Fig. 2J–L. From the GC theory [43]–[45], a vanishing 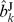 indicates there is no causal influence from neuron 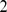 to neuron 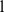, whereas a nonvanishing 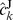 indicates there is a causal influence from neuron 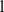 to neuron 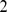. This causal connectivity is consistent with the underlying synaptic connectivity. By definition, the residual cross-correlation 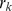 reflects the correlation between the future of neuron 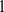 [as embedded in 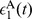] and the history of neuron 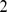 [as embedded in 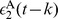], whereas 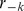 reflects the correlation between the future of neuron 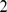 and the history of neuron 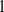. Therefore, 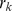 and 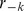 characterize the drive-response relationship between two neurons, as also captured by the regression coefficients 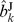 and 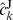 in JR models through Eqs. (12). Furthermore, the GC between two neurons [Eq. (11)] can be approximated by
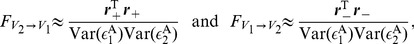
(13)which provide a relation between the GC and the residual cross-correlations.

**Figure 4:**
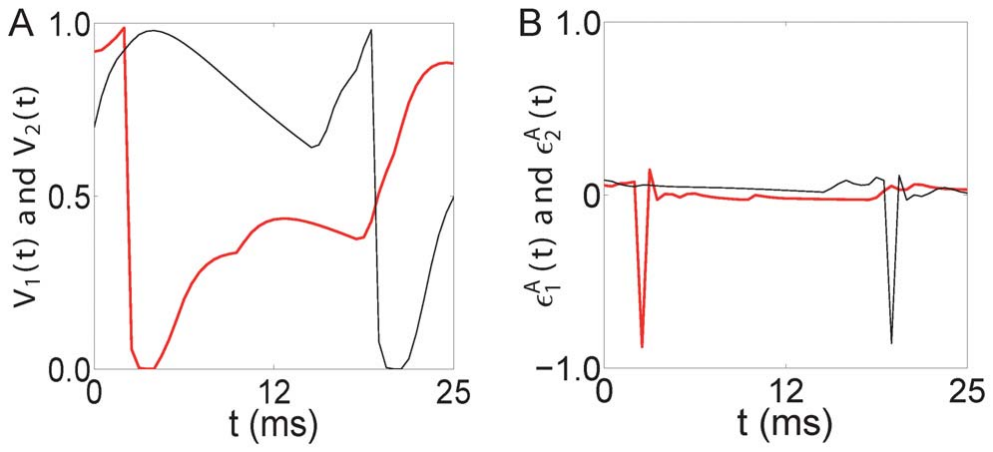
Trajectories of voltages and residuals. For the two-excitatory-neuron network in Fig. 1A, illustrated here are the sample trajectories of voltages in (A) 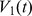 (black online) and 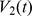 (red online), and corresponding trajectories of residuals in (B) 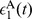 (black online) and 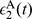 (red online).

Next, we establish the relationship between the STC on residuals and their cross-correlations. Due to the firing-reset dynamics of I
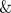
F neurons, the magnitude of 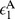 (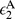) at each 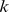
th spike time 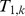 (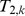) is much larger in absolute value than that at other times as can be seen from Fig. 4. Therefore, the residuals 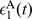 and 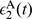 can be approximated in the form of the Dirac delta functions as 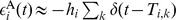, 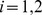, where 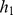 and 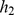 are normalizing factors. Under this approximation, the STC on residuals [Eq. (2)] can be expressed as
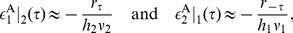
(14)where 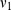 and 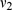 are the firing rate of neuron 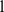 and neuron 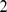, respectively. From Eqs. (13) and (14), it can be seen that the GC 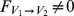 is equivalent to the STC on residual 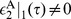, and 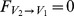 is equivalent to the STC on residual 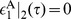 for 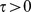. Therefore, the causal connectivity can be well extracted by the nonzero feature of STC on residuals. Note that, as discussed previously, the nonzero feature of STC on residuals is related to the pre-post synaptic connectivity between neurons as shown in Fig. 2G–I. Therefore, we can conclude that the causal connectivity captures well the synaptic connectivity for the I
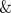
F networks.

Finally, we discuss the relation between GC and the coupling strength 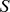 when there exists a synaptic connection between two neurons. Note that the STC 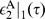 corresponds to the spike-induced change of 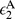. From the I
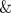
F system [e.g., See Eq. (23) in *Methods*], this change is asymptotically proportional to the coupling strength 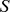 when 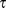 is small. Therefore, combined with Eqs. (13) and (14), we can make a connection that GC is quadratically related to the coupling strength as
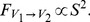
(15)
Fig. 2M–O shows that, in three different dynamical regimes, there is an approximately quadratic relation between GC and the coupling strength, confirming the relationship in Eq. (15). Note that the two-neuron network we discussed above is for the unidirectional case. However, the above analytical derivations are still valid for the case of bidirectional connections.

It is worthwhile to emphasize that it is the 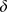-like noise structure of residuals, induced by the firing-reset dynamics, that links STC with the cross-correlation [Eq. (14)]. This is a crucial feature in the I
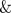
F dynamics that underlies why the GC connectivity can be captured by the STC on whitened signals. The approximate quadratic relationship between GC and 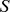 [Fig. 2M-O] ultimately underlies the coincidence between the causal and the structural connectivity for the I
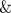
F networks.

### Further investigation of GC

As discussed above, by applying the GC analysis to voltage time series, we have obtained that the synaptic connectivity between neurons can be identified by the GC connectivity for the I
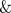
F networks. We now turn to the further investigation of the following issues: (i) whether the synaptic connectivity between a single neuron and a subnetwork or the connectivity between subnetworks can also be revealed by the GC connectivity; (ii) whether the GC connectivity constructed by merely using the spike train time series is also coincident with the synaptic connectivity; (iii) for more realistic neurons, e.g., the exponential I
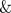
F model, whether there is also a direct connection between synaptic connectivity and GC connectivity; (iv) for networks with both excitatory and inhibitory neurons, whether the network topology can also be successfully reconstructed by the GC analysis.

#### GC connectivity for subnetworks

In extracellular recording, the microelectrode is usually placed away from individual neurons, allowing the activity of a large number of neurons to contribute to the measured signal. We model the signal extracted from such extracellular microeletrodes, i.e., local field potential, by using the voltage averaged over population of neurons and we will term this as the voltage of subnetworks.

For a nine-neuron network [Figs. S1(A) and S2(A)], we can divide it into one subnetwork and one single neuron, where the single neuron corresponds to one neuron in the original network and the subnetwork corresponds to the remaining eight neurons. Through this division, we can construct an effective two-“neuron” that consists of the subnetwork as an effective neuron and the other neuron as another [Fig. S1(C) and S2(C)]. We compute the GC of this effective two-“neuron” network using the voltage of the subnetwork and the voltage of the single neuron [as displayed in Fig. S1(D) and S2(D)]. Our results show that the GC connectivity can successfully capture the structural connectivity between the subnetwork and the single neuron. This reconstruction holds for the case of a subnetwork presynaptic to a single neuron and vice versa (Figs. S1 and S2).

We further examine whether the synaptic connectivity between subnetworks can be revealed by the GC analysis. For a fifteen-neuron network [Fig. S3(A)], we divide this fifteen-neuron network into three subnetworks and construct an effective three-“neuron” network [Fig. S3(C)]. For this effective network consisting of three subnetworks, there are connections that are both “presynaptic” and “postsynaptic” between some subnetworks and there are also “presynaptic” connections only from one subnetwork to another subnetwork. Using voltage time series of these three subnetworks, we compute the GC connectivity [Fig. S3(D)]. Our results show that the GC connectivity is the same as the structural connectivity between subnetworks. From these results, we can conclude that the synaptic connectivity between subnetworks can also be correctly identified by the corresponding GC connectivity.

#### GC connectivity via spike trains

We have so far demonstrated that the GC analysis is effective to reconstruct anatomical connectivity within a network by using continuous-valued signals, e.g., voltage time series. Compared with voltage signals, the recent advent of multiple-electrode recording has made it comparatively easy to simultaneously record spiking activity (action potential) of multiple neurons [65]–[67]. The neuronal activity can often be described by a train of spike events [57], [68], [69],
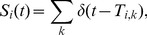
(16)where 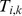 is the 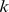
th spike of the 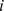
th neuron. The spike train can also be characterized as a binary vector with a component chosen as 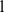 if a spike has occurred in the sample interval, and chosen as 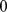 otherwise [70]. Such time series present theoretical challenges because most standard signal processing techniques are designed primarily for continuous-time processes instead of point processes [71].

There are some methods which have already been developed to identify causal relationships between spike trains of simultaneously recorded multiple neurons in experiment. For instance, under the assumption of stationarity, a nonparametric frequency domain approach was proposed to estimate GC directly from the Fourier transforms of spike train data [72]–[74]. Some other statistical methods based on information theory or likelihood function have also been put forth and applied to the analysis of sensory and motor data collected from experiments [75]–[78]. Here, we focus on the time domain GC analysis and study whether the anatomical connectivity of the I
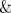
F networks can also be directly mapped to the GC connectivity obtained by using spike train data. Note that, this GC analysis is different than using voltage time series. Unlike voltage data which are continuous-valued data, the spike train data are point-process data and it remains to be determined whether these data can be well described by the multivariate autoregressive models [78].

Following the algorithm of the GC analysis, we use spike train time series (binary vector as described above) to construct the causal connectivity network for the I
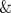
F neuronal systems and compare with their structural connectivity. For the two-neuron network as shown in Fig. 1A, we scan the parameters 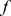 and 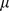 in Poisson input to cover different dynamical regimes and the range of firing rates of realistic neurons. Our results [Fig. S4(A)] show that the synaptic connectivity between two neurons can be well captured by the causal connectivity. For the hundred-neuron network as shown in Fig. 3A, we compute the causal adjacency matrix 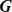 and compare it with the synaptic adjacency matrix 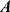. Our results [Fig. S4(B)] again demonstrate that 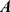 can be successfully reconstructed by 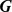 with very high accuracy (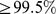). Similarly, as for the case of GC from voltage time series, there is also a gap when we rank the GC by magnitude for all possible directed connections between neurons. Using a horizontal line [(blue online) in Fig. S4(C)] that divides the GC values into two groups, we can obtain 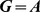 with 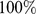 accuracy by using this horizontal line as the GC threshold 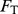.

To demonstrate that our previous analysis of the mechanism underlying the successful reconstruction by using voltage time series for the I
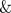
F networks can also be extended to that using spike train time series, we examine the relation between the regression coefficients 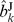, 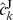 and the residual cross-correlation 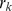, 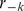 as in Eqs. (12) for the two-neuron network in Fig. 1A. Our results [Fig. S5(A) – (C)] show that the relation [Eqs. (12)] between the regression coefficients and the residual cross-correlation holds very well when the GC connectivity is obtained by using spike train time series. Our results show that there is a vanishing coefficient 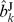, i.e., no causal influence from neuron 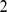 to neuron 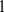, and a nonvanishing 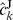, i.e., there is causal influence from neuron 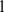 to neuron 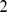. This is also consistent with the synaptic connectivity of the two-neuron network as shown in Fig. 1A. Similarly, due to the 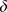-like structure of residuals, we can also obtain that the GC constructed from spike train time series is quadratically related to the coupling strength [as verified in Fig. S5(D) – (F)].

#### GC for exponential integrate-and-fire neuronal networks

To present evidence that our results are not restricted to the standard I
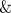
F model [See Eq. (23) in *Methods*], which does not contain spike generation dynamics, we further carry out the GC analysis for the exponential integrate-and-fire (EI
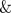
F) neuronal model [See Eq. (24) in *Methods*]. The EI
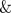
F model captures the action potential of real neurons in a biophysically motivated way by fitting the spike-onset region to realistic neurons, such as the conductance-based Wang-Buzsaki model [79]–[81]. Compared with the standard I
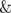
F model which combines linear filtering of input currents with a strict voltage threshold, the EI
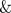
F model allows a replacement of the strict voltage threshold by a relatively realistic smooth spike initiation zone [82], [83]. The model can quite faithfully reproduce response properties of the Hodgkin-Huxley type neurons under rapidly fluctuating inputs [84], [85].

Using the voltage time series obtained by numerically evolving the system of EI
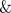
F neurons [See Eq. (24) in *Methods*], we construct regression models for these simulated data and compute causal connectivity of EI
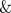
F neuronal networks through the GC analysis. We perform the reconstruction [Fig. S6(A)] for the two-neuron network with the synaptic connectivity shown in Fig. 1A by scanning the parameters 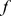 and 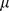. Our results demonstrate that the reconstruction is successful for almost all choices of parameters over different dynamical regimes and with the range over the firing rate (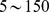
Hz) of real neurons [35], [57]. For the reconstruction of the hundred-neuron network with its synaptic connectivity shown in Fig. 3A, the difference between the synaptic adjacency matrix 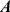 and the constructed causal adjacency matrix 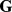 is small [Fig. S6(B)]. We can still obtain a very high accurate reconstruction (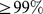). Interestingly, if we rank all GC values in order of magnitude for this hundred-neuron network, as for the case of I
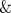
F models, there is also a gap [Fig. S6(C)]. Any horizontal line in the gap [e.g., the blue line in Fig. S6(C)] can be naturally used as a GC threshold 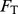 to divide the GC values into two groups, yielding the result 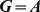 with 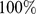 accuracy.

In comparison with the I
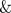
F model, the EI
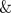
F neuronal model contains an extra spike-generating current term 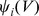 which takes the form of an exponential function. Note that 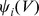 is almost negligible when the voltage of the neuron is below the spike-initiation threshold 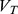. If the neuron fires, 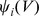 will be dominant and the membrane potential will grow exponentially fast to infinity. After that, the voltage of the neuron will be reset to the reset value. Therefore, the EI
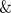
F neuron also possesses the same firing-reset dynamics as the I
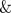
F neuron and our previous analysis, e.g., Eqs. (12) and (15), should also be valid for this more realistic neuronal model. To confirm this, we have verified the relation [Eqs. (12)] between regression coefficients and residual cross-correlations for the two-neuron network in Fig. 1A [as shown in Fig. S7(A) – (C)], and Eqs. (12) is indeed valid for the EI
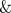
F model. Similarly, as for the case of I
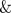
F model, we have a vanishing coefficient 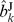, i.e., there is no causal influence from neuron 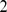 to neuron 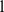, and a nonvanishing 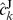, i.e., there is a causal influence from neuron 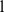 to neuron 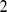. This is again consistent with the underlying synaptic connectivity between the two neurons as shown in Fig. 1A. By using the 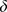-like structure of residuals for the EI
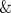
F networks, we can also obtain that GC is quadratically related to the coupling strength as in Eq. (15). This result has also been numerically verified [Fig. S7(D) – (F)].

#### GC for excitatory and inhibitory neuronal networks

**Figure 5:**
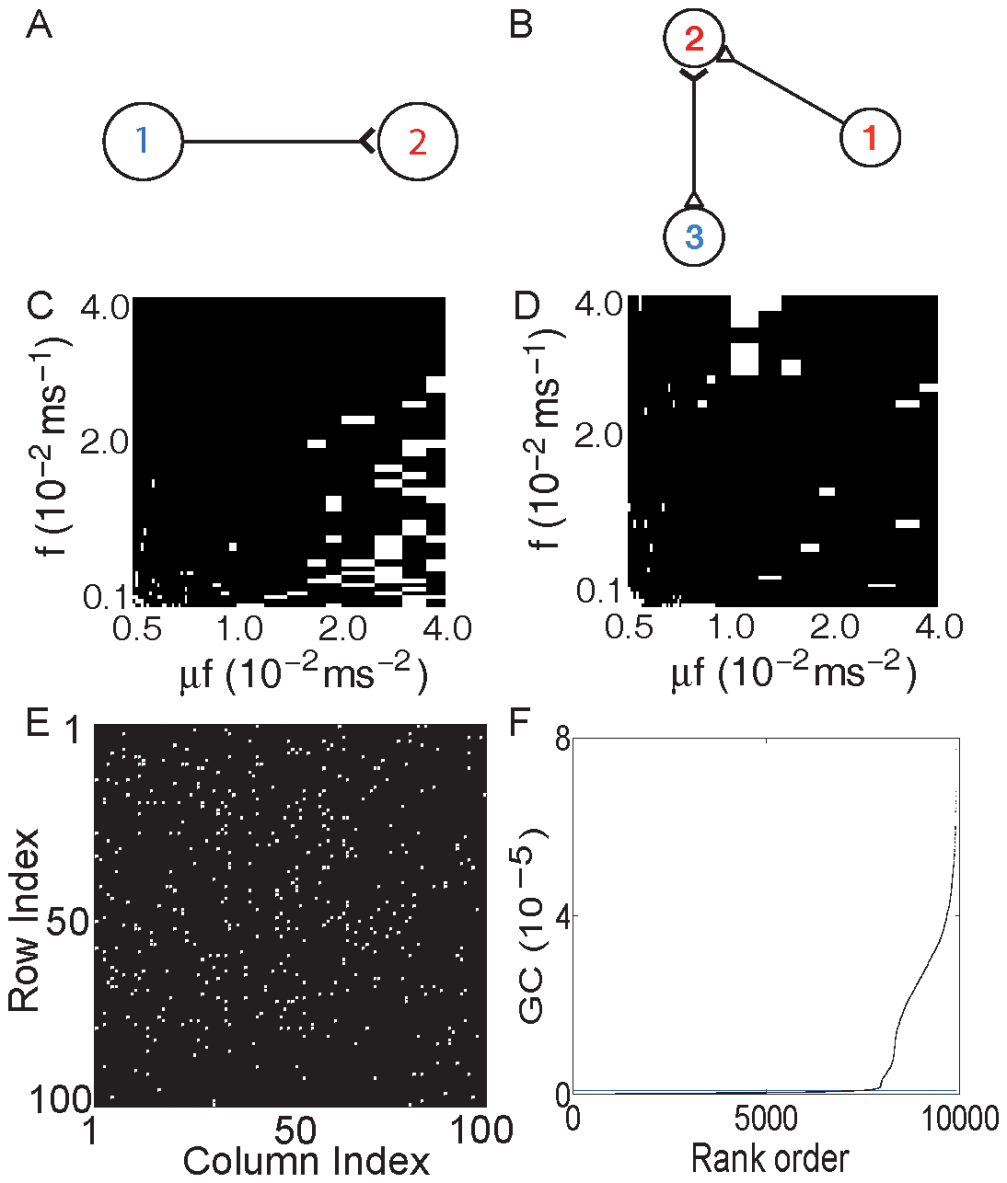
GC connectivity for networks with both excitation and inhibition. Illustrated here are results related to two-neuron and three-neuron I
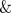
F networks with both excitation and inhibition in (A) – (D), and a large network in (E) and (F). The edge with “
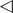
” or “
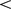
” at the end signifies the directed excitatory or inhibitory connections, respectively. The input parameters are chosen as 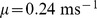 (Poisson input rate) and 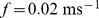 (Poisson input strength). (A) a two-neuron network with one inhibitory neuron (labeled by neuron 1) and one excitatory neuron (labeled by neuron 2). There is only a unidirectional inhibitory synaptic connection from neuron 1 to neuron 2. (B) a three-neuron network with two excitatory neurons (labeled by neuron 1 and 2) and one inhibitory neuron (labeled by neuron 3). There are two excitatory synaptic connections as from neuron 1 to neuron 2 and from neuron 2 to neuron 3. There is also one inhibitory synaptic connection from neuron 3 to neuron 2. (C) The coincidence between 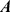 and 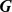 for the two-neuron network in (A). (D) The coincidence between 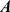 and 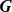 for the three-neuron network in (B). The white color indicates that 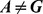, whereas the black color for 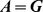. (E) The absolute difference between 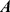 and 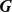, i.e., 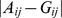 for the large network with 80 excitatory and 20 inhibitory neurons with adjacency matrix shown in Fig. 3A. The white color indicates that 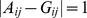, namely, 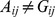 and the black color for 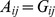. The percentage of total connections (number of nonzero 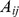) is 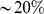 and the average neuronal firing rate is 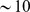
Hz. By significance test (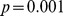, See Text S1 for more details), the total number of 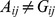 is 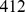 out of 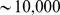 possible pairs of connections. (F) Ranked GC in order of magnitude with the line (blue online) indicating a threshold obtained from the above significance test. Here, the coupling strength from excitatory to excitatory neuron 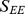 and from excitatory to inhibitory neuron 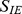 are 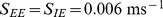 (corresponding EPSP is 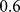
mV), whereas the coupling strength from inhibitory to excitatory neuron 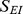 and from inhibitory to inhibitory neuron 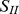 are 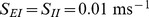 (the corresponding IPSP is 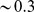
mV).

Finally, we address the issue of whether the above reconstruction can be extended to networks with both excitatory and inhibitory neurons (See Eq. (25) in *Methods*). For a two-neuron network with one excitatory and one inhibitory neurons as shown in Fig. 5A, there is only a unidirectional inhibitory synaptic connection from the inhibitory neuron 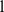 to the excitatory neuron 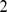. We scan the parameters of Poisson input and compare the synaptic adjacency matrix 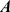 and the constructed causal adjacency matrix 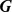. As shown in Fig. 5C, 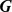 is also highly coincident with 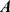 over a wide range of dynamical regimes. For a three-neuron network with two excitatory neurons and one inhibitory neuron as shown in Fig. 5B, there are both excitatory and inhibitory synaptic connections within this small network. By scanning the parameters of Poisson input as shown in Fig. 5D, we also obtain successful reconstruction of the synaptic connectivity 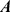 from the causal connectivity 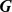 over a wide range of dynamical regimes.

In addition, we have also considered a hundred-neuron network with 80 excitatory and 20 inhibitory neurons. The synaptic connectivity for this hundred-neuron network is chosen to be the same as that in Fig. 3A. The difference between the synaptic adjacency matrix 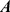 and the constructed causal adjacency matrix 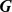 is displayed in Fig. 5E. It can be seen that the accuracy of reconstruction is still very high (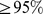). Similarly, we also rank the GC values in order of magnitude and find that, unlike the network with only excitation, there is no clear gap which can naturally divide the GC values into two groups (Fig. 5F). For the GC reconstruction of the network with both excitatory and inhibitory neurons, it is also important to infer the connection type, i.e., excitatory or inhibitory, in addition to the inference of the presence of the connection, and this issue warrants further investigations in the future.

## Discussion

We have shown that the linear GC framework with either continuous voltage or discrete spike train time series, can be successfully applied to the reconstruction of I
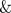
F-type neuronal networks. For such nonlinear networks, the causal connectivity obtained by the GC algorithm with sufficiently long time series corresponds well to their synaptic connectivity. In our simulations, we choose the data length of recording activity to be 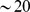
mins [86]–[88] to ascertain that the statistical error is sufficiently small over a wide range of dynamical regimes. However, in real experiments, there may be many complications to maintain the stationarity of neuronal activity with such a long duration of recording [9]. Therefore, we investigate whether the GC reconstruction can be achieved, with high accuracy, within a realistic range of recording length in experiment. For the two-neuron network as shown in Fig. 1, we have investigated how the minimal data length required for GC reconstruction (See Text S1 for more details) using either voltage or spike train time series depends on neuronal firing rate. As shown in Fig. S8(A) and (C), the minimal data length for a successful GC reconstruction by using voltage time series can be as short as 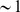
min for both spontaneous firing rate (less than 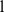
Hz) and the high firing rate range (above 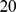
Hz). In contrast, the minimal data length for GC reconstruction by using spike train time series appears to be monotonically dependent on the neuronal firing rate as shown in Fig. S8(B) and (D). When the firing rate is sufficiently high, e.g., above 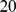
Hz, the required minimal length can be as short as a few seconds. However, if a spontaneous firing rate is sufficiently low, e.g., less than 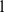
Hz, the minimal data length required for the GC reconstruction can be as long as 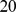
mins. This is somewhat expected because for the GC reconstruction using spike train time series (digital signals), the correlation structure between neurons, as captured by GC influence, can only be reflected by their spikes. If the neuronal firing rate is low, it takes a long time to accumulate a sufficient number of spikes to obtain statistical information of the correlation structure between neurons. However, for the GC reconstruction using voltage time series, the causal influence can be reflected by both subthreshold and suprathreshold (spike) dynamics. Therefore, it may not need that long time to obtain statistical correlation information even if the firing rate is low. Another phenomenon is that the required data length will be shorter if the Poisson input strength becomes smaller. This phenomenon can be clearly seen in Fig. S8 as indicated by the red curve (lower 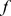), in general, being lower than the blue curve (higher 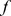). This is also intuitively reasonable since the statistical fluctuations may also decrease when the background input becomes weaker while the coupling strength between neurons is fixed.

**Figure 6:**
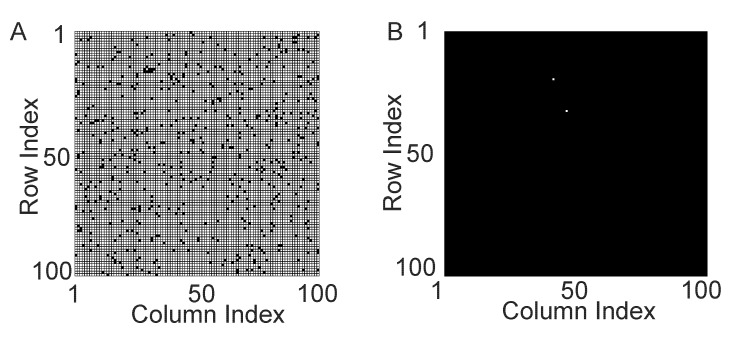
GC reconstruction for a large network with low density connections. Illustrated here are results for a large I
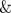
F network (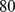 excitatory and 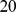 inhibitory neurons) with random connectivity. The indices from 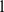 to 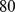 are for excitatory neurons and the indices from 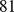 to 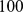 are for inhibitory neurons. The total number of nonzero 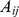 is 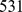 (the percentage of connections is 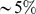) and the average neuronal firing rate is 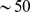
Hz. (A) The synaptic adjacency matrix 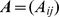 with the white color indicating that 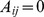 and the black color for 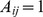. (B) The absolute difference between 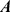 and the causal adjacency matrix 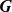, i.e., 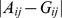. The white color indicates that 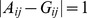, namely, 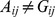 and the black color for 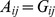. By significance test (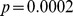, See Text S1 for more details), the total number of 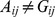 is 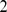 out of 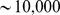 possible pairs of connections. Parameters are chosen as 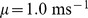 (Poisson input rate), 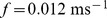 (Poisson input strength), the coupling strength from excitatory to excitatory neuron 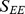 and from excitatory to inhibitory neuron 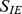 are 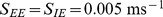 (the corresponding EPSP is 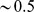
mV), whereas the coupling strength from inhibitory to excitatory neuron 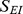 and from inhibitory to inhibitory neuron 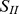 are 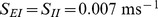 (corresponding to IPSP 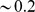
mV).

The computational cost of GC algorithm can be estimated to be 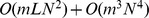, where 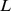 is the data length, 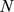 is the total neuron number and 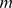 is the regression order in the regression models (See *Methods* for more details). The first term describes the computational cost of covariance matrices and the second term corresponds to the computational cost arising from solving Yule-Walker equations (There are some more efficient algorithms, such as Levinson, Euclidean and Berlekamp-Massey algorithms, which can solve the Yule-Walker equations with 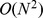 arithmetic operations). Furthermore, we have established a quantitative relationship among the GC, the STC and the coupling strength. Our theoretical analysis based on voltage time series can be naturally extended to the case of spike trains time series and our results show that the GC tool can be directly applied to point-process data [74], [78]. Therefore, the linear GC technique can be potentially used to detect the underlying synaptic connections within a neuronal network by measuring either the voltage trajectories or the spike trains of neurons. We note in passing that the GC reconstruction does not perform well in some cases as shown by the white color region in Fig. 1E–F and Fig. 5C–D. It appears that the statistical error is still not sufficiently small in these cases. We have also examined the dependence of performance of GC reconstruction on the density of the connection matrix. For the case of low density connections (less than 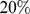) as shown in Figs. 3,5, and 6, the GC reconstruction has a very high accuracy. This indicates the GC reconstruction could potentially be applied to the cortical network reconstruction since many studies have indicated that the structural brain connectivity forms a sparse graph [22], [89]. It appears that for a network of high density connections, e.g., greater than 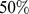, the GC reconstruction does not perform as well, e.g., with approximately 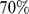 accuracy of reconstruction. We suspect that this could be related to the fact that the signal-to-noise ratio for each pair of coupling to be out of the dense coupling pool is much lower for a network with dense connections than that with sparse connections. A further systematic investigation is warranted to achieve a full understanding of this issue in the future.

In addition, we have shown that the synaptic connection in some coarse-grained sense, e.g., the connection between an individual neuron and a subnetwork, or the connection between subnetworks, can also be recovered through GC analysis. In our work, the recorded time series of a subnetwork is the voltage response averaged over neurons within the subnetwork, which can be viewed as a model for the local field potential (LFP). Note that the LFP in our case includes both the subthreshold dynamics and the spike-reset dynamics, and it may be different from the LFP normally measured in experiment, which contains only a low-pass filtered component of population voltages [90]. As for the averaged voltage response processed by a low-pass filter, our conclusions can still be valid as long as the low-pass filter is chosen to be a causal filter, namely, the filter output depends only on past and present inputs. The reason can be explained as follows: if the averaged voltage response (AVR) is processed by a causal low-pass filter, then the transformation between the filtered AVR and the original AVR is linear and invertible. The GC is invariant under such filtering because of the invariance of GC under invertible linear transformation [44], [45]. In fact, the filtered AVR is different from the original AVR. However, the residual of auto regression for the filtered AVR is only different from that for the original AVR by a factor if the filter is chosen to be a causal filter. Therefore, the corresponding structure of both the residual cross-correlation and the STC on residuals are the same as those of the original AVR (although the amplitude may be different by a factor). As a result, our conclusions about network reconstruction and our theoretical analysis remain valid.

There are other important issues that remain to be fully elucidated in the future. One of them is whether an accurate reconstruction can still be obtained when the inputs to neurons are correlated. For instance, it is quite common that a pair of neurons may receive a common synaptic input from another neuron [91]–[93]. Our study shows that an approximate reconstruction can be achieved (with an accuracy greater than 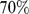) if the correlation coefficient for two input spike trains is less than 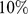 [94]. As an illustration of how the success of GC reconstruction depends on the input correlation, we have studied the effects of input correlation on the GC reconstruction by using either voltage or spike train time series for the two-neuron network as shown in Fig. 1A. In addition to its own Poisson input (independent of each other) with the same rate 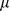 and the same strength 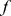, each neuron in the network receives a common Poisson input with rate 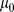 and strength 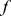. The percentage of common Poisson input 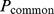 is defined by 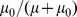. As discussed previously, a successful GC reconstruction for the synaptic connectivity of this network indicates that the GC from neuron 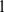 to neuron 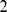 (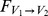 or 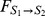) is significantly nonzero, whereas the GC from neuron 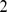 to neuron 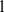 (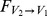 or 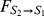) nearly vanishes. Therefore, the GC ratios 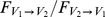 and 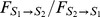 can be used as a measure of quantifying how successful GC reconstructions are. As shown in Fig. S9, we plot such GC ratios as a function of 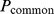. It can be seen from Fig. S9 that the magnitude of both GC ratios drops rapidly as 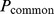 increases, thus indicating that the GC reconstruction eventually fails when 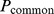 is large. However, as shown in Fig. S9, the GC reconstruction can still be trusted if the percentage of common Poisson input is less than 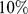 because there is about one order of magnitude difference between 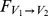 (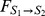) and 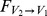 (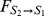).

Another issue is related to the synchronization among neurons [95]. We have found that, for a nearly (not fully spike-to-spike) synchronized regime, the reconstruction can be achieved by refining sampling. It is obvious that the drive-response scenario, which the GC theory addresses, would be difficult to disentangle when the neuronal network falls into the spike-to-spike synchronized dynamical regime [95]–[98]. In such cases, the causal influences between neurons would decrease, whereas the instantaneous causality would increase [14]. As for the sampling rate used in our simulations, we choose 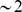
kHz for our sampling rate (the time scale of refractory period in our neuronal models is 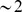
ms, therefore, the sampling rate should be chosen larger than 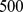
Hz to capture this time scale). In addition, we have also examined different sampling rates between 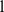
kHz and 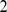
kHz and found that the structural connectivity can always be revealed by GC connectivity with similar high accuracy.

Finally, we point out that there are some other methods that have been developed to reconstruct the network topology, e.g., phase resetting or chaotic synchronization [99]–[107]. These techniques were applied to either coupled oscillators or current-based networks [108], which can be regarded as the reduced form of the general conductance-based I
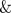
F networks. For instance, in the limit 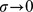, 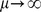 and 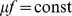, the conductance-based I
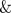
F network reduces to Mirollo-Strogatz oscillators which are widely used in the study of synchronization phenomena [109]. Therefore, our work provides a general methodology to reconstruct the network topology for conductance-based I
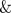
F networks. In terms of the GC theory, there are also some extensions to investigate causal relationship for nonlinear and non-Gaussian time series, e.g., the kernel-Granger causality method [110], [111]. The concept of such nonlinear GC is formulated by using the theory of reproducing kernel Hilbert spaces that are spanned by choosing proper kernel functions. The form of kernel functions relies on the nonlinearity of the original dynamical systems, which is usually unknown. In our work, if we choose the kernel function for I
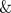
F networks to be a bilinear function, then the nonlinear GC framework reduces to the linear GC framework. Our results have shown that such reduced nonlinear GC analysis is able to capture well the underlying topology of I
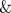
F networks.

## Methods

### Granger Causality (GC) Analysis

We first recall theoretical definitions of GC for time series in the bivariate case and the conditional GC for time series in the multivariate case (In the discussion of GC, we will always assume that the mean of time series has been subtracted and the expectations of time series for both bivariate and multivariate cases are zero). The idea of GC was formalized in the context of linear regression models [43], [112]. Specifically, if the variance of the prediction error of the first time series in the auto regressive model is reduced by incorporating the knowledge of the second one, then the second time series is said to have a causal influence on the first one [44], [45]. The roles of the two time series can be reversed to address the question of causal influence in the opposite direction. The GC theory has been widely applied to many research fields as mentioned in *Introduction* [14], [46].

#### Bivariate case

For two time series 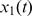 and 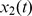, their auto regression (AR) models can be represented by
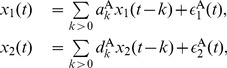
(17)where 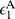 and 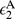 are residuals (prediction errors) of AR processes for 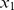 and 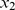, respectively. To illustrate GC relations between 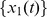 and 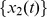, we further consider their joint regression (JR) models as
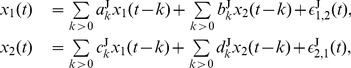
(18)where 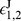 is the residual of JR process for 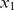 by further incorporating the history of 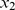, and 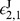 is the residual of JR process for 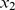 by further incorporating the history of 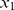 [14], [113]. By assuming that 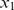 and 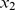 are wide-sense stationary, i.e., their means and variances are constants, the GC from 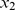 to 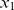, denoted by 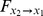, and that from 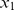 to 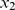, denoted by 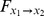 are defined as
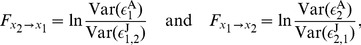
(19)where 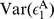 and 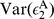 are the variances of the residuals 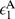 and 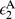 in AR models, respectively. These variances quantify the accuracy of the autoregressive prediction of 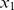 and 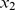 at the present moment based on their own past. The quantities 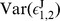 and 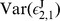 are the variances of the residuals 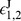 and 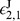 in JR models, respectively. They represent the accuracy of predicting the present value of 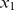 or 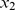 based on the previous values of both 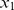 and 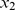 [44], [45]. For instance, if 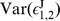 is less than 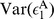, then there is an improvement in the prediction of 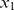 by incorporating the history of 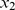, thus 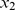 is said to have a causal influence on 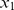. Note that, both 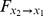 and 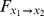 cannot be negative by definition. In particular, 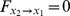 [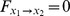] corresponds to the situation where there is no causal influence from 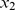 [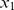] to 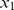 [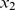] [14], [44].

#### Multivariate case

In the case of multivariate time series 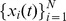 (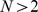), the causal relation between two time series, say, 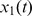 and 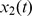, can be directly mediated or it can be indirectly mediated by a third one, say 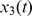. However, the above pairwise analysis for the bivariate case cannot distinguish whether the causal influence between 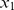 and 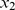 is direct or indirect [14]. The framework of conditional Granger causality was developed to address such situations [45]. The procedure can be carried out as follows: for any two time series 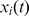 and 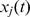 among the set 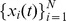, the conditional AR processes are represented by
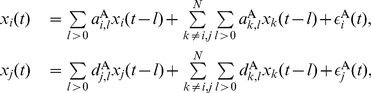
(20)where “conditional” means the auto regressions of 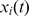 and 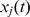 are performed when the history of all other time series 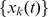 (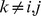) is given. Furthermore, we consider the conditional JR processes for 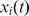 and 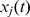 as
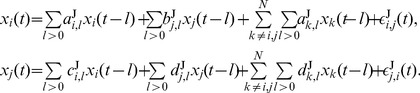
(21)From Eqs. (20) and (21), the conditional GC from 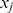 to 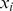, denoted by 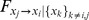, and that from 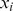 to 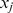, denoted by 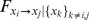, are defined as
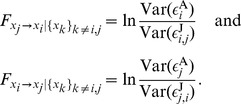
(22)Note that, in Eqs. (20) and (21), both the auto and joint regressions of 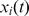 and 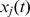 are performed by including the history of other time series 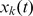 (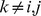). Therefore, if the causal influence between 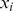 and 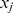 is entirely mediated by some other time series in the set 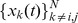, the variance of residuals in conditional AR models will be equal to the variance of residuals in conditional JR models, i.e., 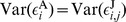 and 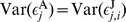. Therefore, we have 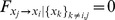 and 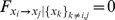, that is, no further improvement in the prediction of 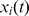 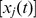 in JR models can be expected by including past measurements of 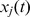 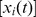. On the other hand, if the causal relation between 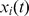 and 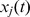, say from 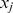 to 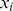, is direct, the inclusion of past measurements of 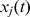 in addition to that of 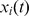 and 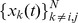 will result in a better prediction of 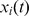, thus leading to 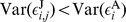 and 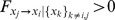.

### GC algorithm for I
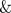
F networks

Here, for an I
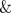
F network with 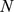 neurons, we propose the following numerical algorithm of computing GC through the voltage time series of neurons (similarly for the case of using spike train time series). We denote the voltage trajectory of the 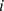
th neuron by 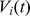, and the GC from the 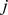
th neuron to the 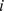
th neuron, obtained from these voltage time series, by 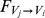. According to the above definition of GC, we compute both regression residuals 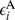 and 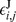 as in Eqs. (20) and (21) to obtain 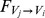 in Eq. (22). Note that the GC from the 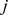
th neuron to itself is always zero by definition (For neuronal systems discussed in our work, we do not consider autapses in the network). The flow of the algorithm to compute each 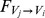 for every pair of neurons, where 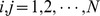 and 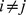, can be described as follows (See Text S1 for more details):

**Step 1**: Evolve the I
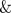
F network dynamics [e.g., Eq. (23) in *Methods*] numerically and record the voltage signal averaged over each small time window (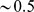
ms, i.e., sampling rate is 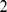
kHz) to form voltage time series of 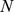 neurons as 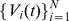, 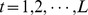. In most of our simulations, 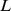 is chosen to be 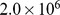, which corresponds to the length of time series 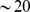
mins. Then, construct an 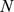 dimensional vector 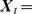 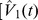, 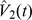, 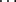, 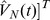 and 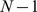 dimensional vectors 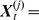 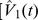, 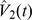, 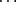, 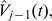 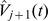, 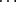, 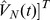 for 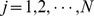, where 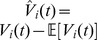 has zero mean and the superscript 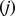 in 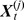 denotes the fact that the 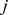
th component 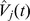 in 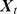 is removed.

**Step 2**: For any given regression order 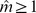, compute the covariance matrix functions 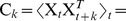 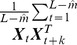 for 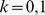, 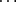, 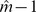 to construct the Yule-Walker equations. We denote the coefficient matrix and the right hand side of the Yule-Walker equations by 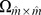 and 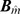, respectively [Eqs. (4) – (7) in Text S1]. Solve the Yule-Walker equations to obtain the regression coefficients, which are denoted by 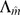 [Eq. (6) in Text S1]. Then, substitute 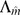 into the regressive equations [Eq. (1) in Text S1] to obtain the residual vector 
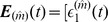
, 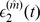, 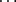, 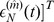 and calculate its covariance matrix, which is denoted by 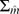 [Eq. (2) in Text S1].

**Step 3**: Now, for each 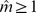, we have 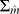, therefore, we can obtain the BIC function [Eq. (10) in Text S1] as a function of 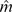. Use the BIC criterion to find the correct regression order 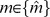 which corresponds to the situation where the BIC function reaches its minimum.

**Step 4**: Then, choose 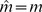 and return to Step 2 to obtain the residual vector 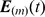 and also the covariance matrix 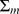. The 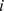
th diagonal element of 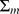, denoted by 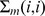, corresponds to 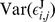, which is the variance of the residual 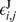 in the joint regression model of the 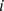
th neuron by incorporating the information of the 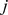
th neuron (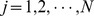) in addition to all other 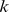
th neurons (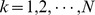 and 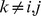). Set 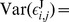 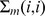, for different 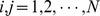.

**Step 5**: Let 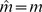 and use 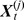 for each 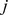 (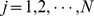) to follow the procedure of Step 2 to obtain the residual vector 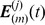 and the covariance matrix 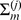. If the neuron index 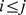, the 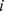
th diagonal element 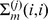 corresponds to 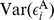, which is the variance of the residual 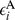 in the AR model of the 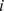
th neuron by incorporating the information of all other neurons except the 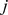
th neuron, set 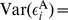 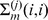. Otherwise, for 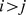, the 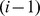
th diagonal element 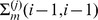 corresponds to 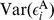 and set 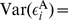 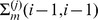.

**Step 6**: Compute all the GC values 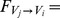 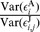 for all pairs of neurons, i.e., 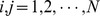.

### Integrate-and-fire (I
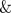
F) neuronal network

We consider an I
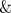
F network with 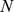 conductance-based, pulse-coupled, excitatory point neurons. Under a Poisson drive, its dynamics is governed by
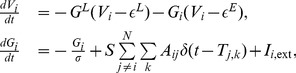
(23)where the index 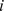 labels the 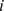
th neuron in the network. 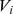 is the membrane potential and 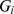 is the excitatory synaptic conductance. 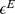 is the excitatory reversal potential. 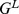 and 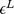 are the leaky conductance and the leaky reversal potential, respectively. The excitatory synaptic dynamics are described by 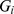, which rises instantaneously upon receiving a spike and has a decay time scale 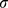. The voltage of the 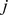
th neuron 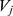 evolves continuously according to Eq. (23) until it reaches the firing threshold 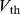, at which point the 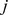
th neuron is referred to as producing an action potential or emitting a spike (the time of the 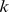
th spike is recorded as 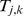). Then, this spike triggers postsynaptic events in all the neurons that the 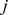
th neuron is presynaptically connected to and changes their conductances with the coupling strength 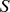 [the corresponding physiological excitatory postsynaptic potential (EPSP) is 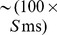
mV]. Here, the synaptic connectivity of the network is characterized by an adjacency matrix 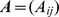, where 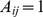 (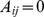) means the presynaptic 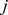
th neuron is connected (unconnected) to the postsynaptic 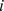
th neuron. Meanwhile, 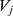 after the 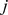
th neuron's spike is reset to the reset voltage 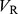 and is held at 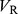 for an absolute refractory period of 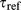 ms. Each neuron (say, the 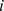
th neuron) in the system is also driven by a stochastic feedforward input 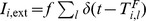, a spike train sampled from a Poisson process with rate 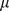. We denote 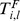 as the 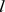
th spike from the feedforward input to the 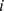
th neuron and the delta function associated with this spike instantaneously increases the 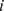
th neuron's conductance by magnitude 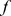.

In comparison with the classical Hodgkin-Huxley (HH) neuronal model with detailed ionic currents to resolve the stereotypical spike dynamics [114], the model (23), as a reduced neuronal model, is much more efficient in terms of computation while capturing sufficiently rich network dynamics of HH models [58], [59], [96], [115], [116]. Therefore, it has been widely used in large-scale simulations to address information processing issues arising from neuronal systems [36]–[42]. In the reduced-dimensional units, in which only time retains dimensional, with units of conductance being [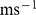], the parameters in the model (23) are chosen as [117]: 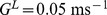, 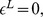 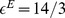, 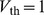, 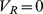, 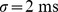, 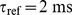, which correspond to typical physiological values: 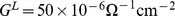, 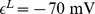, 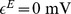, 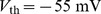.

### Exponential integrate-and-fire (EI
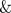
F) neuronal network

The dynamics of an excitatory EI
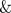
F neuronal network with 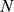 neurons is governed by
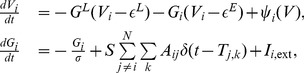
(24)where the function 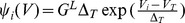 characterizes the spike-generating current of the 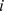
th neuron [80]. Here, 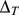 is the slope factor and 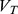 is the spike-initiation threshold potential. Each neuron (say, the 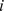
th neuron) in the system is driven by an external stochastic feedforward input as 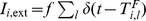. If the input current exceeds some threshold 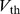, the membrane potential of the 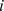
th neuron 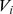 will diverge to infinity in finite time since 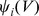 is supralinear. This divergence is identified as the emission of a spike of the 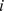
th neuron. And at the same time 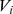 is reset to the reset voltage 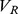 and is held at 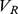 for an absolute refractory period of 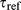 ms. Note that, with 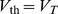, the EI
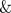
F model reduces to the standard I
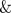
F model under the limit 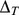 goes to zero. Some parameters are chosen to be the same as those in I
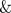
F models and others in the reduced-dimensional units in the model (24) are chosen as [80], [84]: 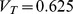, 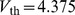, 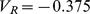, 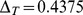.

### I
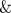
F networks with both excitation and inhibition

For a conductance-based I
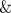
F network with 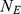 excitatory neurons and 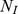 inhibitory neurons, its dynamics under Poisson drives is governed by
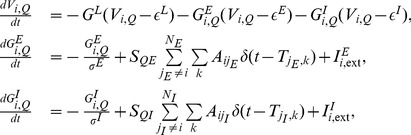
(25)where the 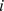
th neuron with type 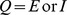, has both excitatory conductance 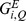 and inhibitory conductance 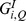, and the 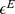 and 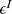 are the excitatory and inhibitory reversal potentials, respectively. If an excitatory neuron (say, the 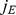
th neuron) fires and is presynaptic to the 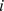
th neuron (i.e., 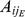 = 1), the 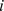
th neuron's excitatory conductance 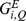 will be increased by the coupling strength 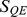. If an inhibitory neuron (say, the 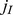
th neuron) fires and is presynaptic to the 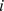
th neuron (i.e., 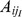 = 1), the 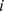
th neuron's inhibitory conductance 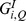 will be increased by the coupling strength 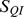. [the corresponding physiological inhibitory postsynaptic potential (IPSP) is 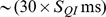
mV]. The 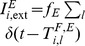 and 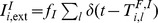 are external Poisson drives for the 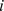
th neuron arising from background excitation and inhibition, respectively. For simplicity, we only consider the excitatory background input, i.e., 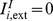, and choose the input rate 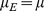 and input strength 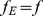. In the reduced-dimensional units, the parameters of inhibition in the model (25) are chosen as [117] 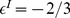, 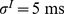, which correspond to typical physiological values, 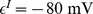. Other parameters are chosen to be the same as those in excitatory I
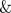
F models [Eq. (23)].

## Supporting Information

Figure S1
**GC from a subnetwork to a single neuron.** For this nine-neuron network, we can divide it into one subnetwork and one single neuron, where the single neuron corresponds to neuron 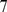 and the subnetwork corresponds to the remaining eight neurons. Here, the subnetwork is presynaptic to the single neuron. Parameters are chosen as 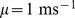 (Poisson input rate), 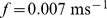 (Poisson input strength), and the coupling strength 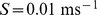 (the corresponding EPSP is 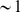
mV). (A) A nine-neuron network with its synaptic connectivity. (B) The constructed causal adjacency matrix 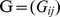 which captures the synaptic connectivity in (A). The white color in 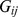 means there is no causal connection from neuron 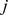 to neuron 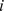, i.e., 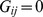, and the black color represents the existence of a causal connection from neuron 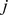 to neuron 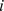, i.e., 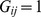. (C) An effective two-neuron network constructed from (A), where the “neuron 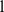
” represents the subnetwork that consists of all neurons in (A) except for neuron 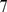 as indicated by gray boxes (red online) in (B). The neuron 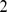 represents neuron 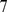 in (A). The voltage of “neuron 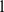
” is the mean voltage averaged over all neurons in the subnetwork. (D) The computed causal adjacency matrix for (C), which successfully captures the unidirectional connection from the subnetwork “neuron 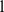
” to the single neuron labeled by 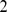 [neuron 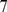 in (A)].(EPS)

Figure S2
**GC from a single neuron to a subnetwork.** For this nine-neuron network, we can divide it into one subnetwork and one single neuron, where the single neuron corresponds to neuron 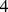 and the subnetwork corresponds to the remaining eight neurons. Here, the subnetwork is postsynaptic to the single neuron. Parameters are chosen as 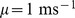 (Poisson input rate), 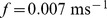 (Poisson input strength), and the coupling strength 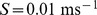 (corresponding to EPSP 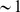
mV). (A) A nine-neuron network with its synaptic connectivity. (B) The constructed causal adjacency matrix 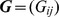 which captures the synaptic connectivity in (A). The white color in 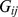 means there is no causal connection from neuron 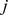 to neuron 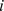, i.e., 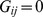, and the black color represents the existence of causal connection from neuron 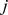 to neuron 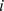, i.e., 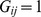. (C) An effective two-neuron network constructed from (A), where neuron 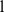 represents neuron 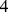 in (A), “neuron 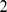
” represents the entire network (A) except for neuron 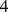. The voltage of “neuron 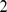
” is the voltage averaged over all neurons in the subnetwork, as indicated by gray boxes (red online). (D) The computed causal adjacency matrix for (C), which captures the unidirectional causal influence from the single neuron, labeled by 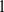 [neuron 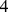 in (A)], to the subnetwork “neuron 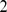
”.(EPS)

Figure S3
**GC between subnetworks.** To construct subnetworks, we divide this fifteen-neuron network into three subnetworks and construct an effective three-“neuron” network. Parameters are chosen as 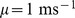 (Poisson input rate), 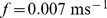 (Poisson input strength), and the coupling strength 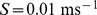 (corresponding to EPSP 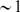
mV). (A) The synaptic adjacency matrix 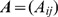 for a fifteen-neuron network. The white color in 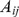 indicates that there is no synaptic connection from neuron 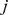 to neuron 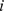, i.e., 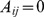 and the black color represents that the neuron 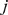 is presynaptic to the neuron 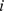, i.e., 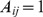. (B) The causal adjacency matrix 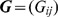 constructed by GC, which is identical to 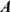. (C) An effective three-neuron network constructed from (A), where the voltage of neuron “
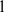
”,“
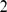
” and “
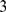
” are the averaged response over each group of neurons, respectively. “Neuron 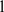
” [indicated by the red box in (A)] represents a subnetwork from neuron 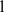 to neuron 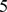, “neuron 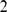
” [indicated by the blue box in (A)] represents a subnetwork from neuron 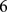 to neuron 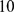, and “neuron 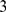
” [indicated by the green box in (A)] represents a subnetwork from neuron 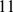 to neuron 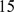. (D) The computed causal adjacency matrix for (C), which captures the effective synaptic connections between the subnetworks.(EPS)

Figure S4
**GC connectivity using spike train.** Network reconstruction by GC using the spike train time series of the I
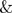
F model. (A) The coincidence between the synaptic adjacency matrix 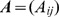 and the causal adjacency matrix 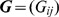 for the two-neuron network in Fig. 1A. The white color indicates that 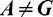, and the black color for 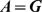. (B) The absolute difference between 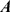 and 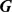, i.e., 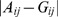, for the hundred-neuron network in Fig. 3A. The white color indicates that 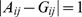, namely, 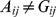 and the black color for 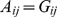. The total number of 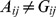 is 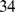 out of 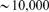 possible pairs of connections (with 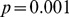 in the significance test). (C) Ranked GC in order of magnitude for the hundred-neuron network in Fig. 3A. The gray line (blue online) indicates a threshold in the gap of the ranked GC. Parameters are chosen as 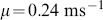 (Poisson input rate), 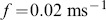 (Poisson input strength), and the coupling strength 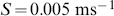 (corresponding to EPSP 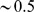
mV).(EPS)

Figure S5
**GC analysis using spike train.** Illustrated here is the validity of the relations used in the mechanism analysis computed by using the spike trains of the two excitatory neurons of the I
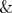
F network in Fig. 1A with different Poisson input rate 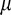 for the highly fluctuating regime [(A) and (D)] with 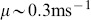, intermediate regime [(B) and (E)] with 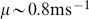 and mean-driven regime [(C) and (F)] with 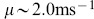. The fixed input strength 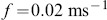. (A), (B), and (C) are regression coefficients 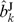 (blue “plus” online), 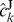 (red “cross” online) and their approximations 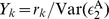 (“square” symbol), 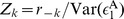 (“circle” symbol) for three different dynamical regimes. (D), (E), and (F) are the GC 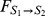 (red “star” online) as a function of coupling strength 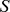 for three different dynamical regimes. The line (black online) is a quadratic fit.(EPS)

Figure S6
**GC connectivity for EI** 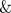 **F networks.** Network reconstruction by GC using the voltage time series of the EI
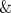
F model. (A) The coincidence between the synaptic adjacency matrix 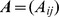 and the causal adjacency matrix 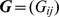 for the two-neuron network in Fig. 1A. The white color indicates that 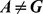, and the black color for 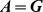. (B) The absolute difference between 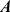 and 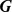, i.e., 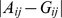, for the hundred-neuron network in Fig. 3A. The white color indicates that 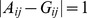, namely, 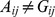 and the black color for 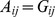. The total number of 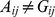 is 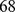 out of 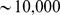 possible pairs of connections (with 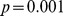 in the significance test). The average neuronal firing rate is 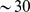
Hz. (C) Ranked GC in order of magnitude for the hundred-neuron network in Fig. 3A. The gray line (blue online) indicates a threshold in the gap of the ranked GC. Parameters are chosen as 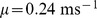 (Poisson input rate), 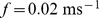 (Poisson input strength), and the coupling strength 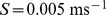 (corresponding to EPSP 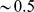
mV).(EPS)

Figure S7
**GC analysis for EI** 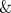 **F networks.** Illustrated here is the validity of the relations in the mechanism analysis of GC computed by using the voltage time series of the two excitatory neurons of the EI
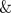
F network in Fig. 1A with different Poisson input rate 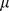 for the highly fluctuating regime [(A) and (D)] with 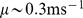, intermediate regime [(B) and (E)] with 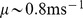 and low fluctuating regime [(C) and (F)] with 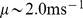. The fixed input strength 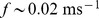. (A), (B), and (C) are regression coefficients 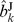 (blue “plus” online), 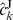 (red “cross” online) and their approximations 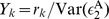 (“square” symbol), 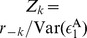 (“circle” symbol) for three different dynamical regimes. (D), (E), and (F) are the GC 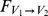 (red “star” online) for three different dynamical regimes. The line (black online) is a quadratic fit.(EPS)

Figure S8
**Minimal data length vs. neuronal firing rate.** Illustrated here is the minimum data length required for GC reconstruction of the I
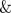
F network in Fig. 1A as a function of neuronal firing rate. (A) GC reconstruction using voltage time series with firing rate between 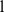
Hz and 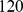
Hz. (B) the same as (A) but using spike train time series. (C) the same as (A) but with firing rate between 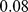
Hz and 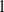
Hz. (D) the same as (B) but with firing rate between 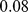
Hz and 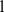
Hz. The Poisson input strength 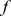 is chosen as 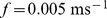 [red “star”, the line is for guiding the eye only.], 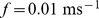 [blue “circle”, the line is for guiding the eye only.] and the coupling strength 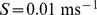 (corresponding to EPSP 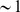
mV). The Poisson input rate 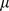 is chosen to satisfy the corresponding neuronal firing rate.(EPS)

Figure S9
**GC ratio vs. the percentage of common Poisson input.** Illustrated here are the GC ratios 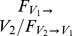 and 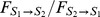 for the I
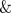
F network in Fig. 1A. Neuron 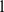 and neuron 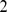 are driven by two independent background Poisson inputs with same rate 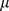 and same strength 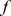. In addition, both neurons are also driven by another common Poisson input (independent of their background Poisson input) with rate 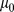 and strength 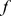. The percentage of common Poisson input 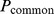 is defined by 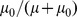. (A) The ratio of computed GC by using voltage time series: 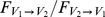 as a function of 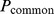. (B) The ratio of computed GC by using spike train time series: 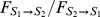 as a function of 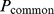. In (A) and (B), the red “star” symbol linked by solid line corresponds to the highly fluctuating regime with 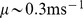 as shown in Fig. 2A, the blue “circle” symbol linked by solid line corresponds to the intermediate regime with 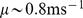 as shown in Fig. 2B and the black “square” symbol linked by solid line corresponds to the mean-driven regime with 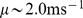 as shown in Fig. 2C. Parameters in (A) and (B) are chosen as 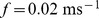 [red “star”, the line is for guiding the eye only.], 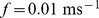 [blue “circle”, the line is for guiding the eye only.], 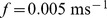 [black “square”, the line is for guiding the eye only.] and the coupling strength 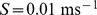 (corresponding to EPSP 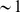
mV).(EPS)

Text S1 **Computational issues of GC.**
(PDF)
