# Supplementary material for: Granger Causality Network Reconstruction of Conductance-Based Integrate-and-Fire Neuronal Systems
Source: PLoS One. 2014 Feb 19;9(2):e87636. doi: 10.1371/journal.pone.0087636 (PMC3929548; doi:10.1371/journal.pone.0087636)
Supplement: Text S1 — Computational issues of GC. (PDF) [file pone.0087636.s010.pdf]

Supporting Information

Granger Causality Network Reconstruction of Conductance-based Integrate-and-Fire Neuronal Systems

Douglas Zhou, Yanyang Xiao, Yaoyu Zhang, Zhiqin Xu, David Cai

Computational issues of GC

In the theoretical definition of GC, one assumes that the data length of time series and the regression order in autoregressive processes are both infinite [1]. In practice, however, the measured time series in experiment has only finite length. With finite-length data, some important issues have to be addressed. The first issue is how to choose a correct regression order since the total length of data is finite. It has been shown that an improper choice of regression order can cause large errors in linear regression models [2, 3]. The second issue is to determine whether the causal influence between time series is statistically significant or arises from statistical error introduced by finite-length data and finite regression orders in the numerical computation of GC.

In the following, we discuss the methodology to address the above issues along with the numerical algorithm of evaluating GC for a given set of empirical time series $\{x_i(t)\}_{i=1}^N$. Let $\boldsymbol{X}_t = [\hat{x}_1(t), \hat{x}_2(t), \cdots, \hat{x}_N(t)]^T$ be an N dimensional time series with L being the length for simultaneously recorded $\{x_i(t)\}_{i=1}^N$. Here, the notation of superscript T in $\boldsymbol{X}_t$ represents matrix transposition and $\hat{x}_i(t) = x_i(t) - \mathbb{E}[x_i(t)]$ has a zero mean. Assuming that $\boldsymbol{X}_t$ is stationary, for a given regression order $\hat{m}$, we can construct the following regressive equations [2, 3]

$$\boldsymbol{X}_t + \boldsymbol{A}_1\boldsymbol{X}_{t-1} + \cdots + \boldsymbol{A}_{\hat{m}}\boldsymbol{X}_{t-\hat{m}} = \boldsymbol{E}_{(\hat{m})}(t), \tag{1}$$

where $\boldsymbol{A}_i$ are $N \times N$ regression-coefficient matrices ($i = 1, 2, \cdots, \hat{m}$) and $\boldsymbol{E}_{(\hat{m})}(t) = [\epsilon_1^{(\hat{m})}(t), \epsilon_2^{(\hat{m})}(t), \cdots, \epsilon_N^{(\hat{m})}(t)]^T$ is a vector of residuals which have zero mean. The covariance matrix of $\boldsymbol{E}_{(\hat{m})}(t)$, denoted by $\boldsymbol{\Sigma}_{\hat{m}}$, is given by

$$\boldsymbol{\Sigma}_{\hat{m}} = \langle \boldsymbol{E}_{(\hat{m})}(t)\boldsymbol{E}_{(\hat{m})}^T(t)\rangle. \tag{2}$$

Note that the correlation between $\boldsymbol{E}_{(\hat{m})}(t)$ and $\boldsymbol{X}_{t-k}$ (for any $1 \le k \le \hat{m}$) is zero. Therefore, by multiplying $\boldsymbol{X}_{t-k}^T$ ($k = 1, 2, \cdots, \hat{m}$) on both sides of Eqs. (1) and then taking expectations, we can obtain the Yule-Walker equations as follows [2, 3]

$$\boldsymbol{C}_{-k} + \boldsymbol{A}_1\boldsymbol{C}_{-k+1} + \cdots + \boldsymbol{A}_{\hat{m}}\boldsymbol{C}_{-k+\hat{m}} = 0, \quad 1 \le k \le \hat{m}, \tag{3}$$

where $\boldsymbol{C}_\tau = \langle \boldsymbol{X}_t\boldsymbol{X}_{t+\tau}^T\rangle$ is the covariance matrix of $\boldsymbol{X}_t$ and $\boldsymbol{C}_{-\tau} = \boldsymbol{C}_\tau^T$ for $\tau \ge 0$.

To obtain the regression coefficients $\boldsymbol{A}_1$, $\boldsymbol{A}_2$, $\cdots$, $\boldsymbol{A}_{\hat{m}}$, we rewrite Eqs. (3) as

$$\begin{pmatrix} \boldsymbol{C}_0 & \boldsymbol{C}_1^T & \cdots & \boldsymbol{C}_{\hat{m}-1}^T \\ \boldsymbol{C}_1 & \boldsymbol{C}_0 & \cdots & \boldsymbol{C}_{\hat{m}-2}^T \\ \vdots & \vdots & \ddots & \vdots \\ \boldsymbol{C}_{\hat{m}-1} & \boldsymbol{C}_{\hat{m}-2} & \cdots & \boldsymbol{C}_0 \end{pmatrix} \begin{pmatrix} \boldsymbol{A}_1^T \\ \boldsymbol{A}_2^T \\ \vdots \\ \boldsymbol{A}_{\hat{m}}^T \end{pmatrix} = - \begin{pmatrix} \boldsymbol{C}_1 \\ \boldsymbol{C}_2 \\ \vdots \\ \boldsymbol{C}_{\hat{m}} \end{pmatrix} \tag{4}$$

For simplicity, we denote the symmetrical coefficient matrix of linear equations (4) by

$$\boldsymbol{\Omega}_{\hat{m}\times\hat{m}} = [\boldsymbol{\Omega}_{ij}]_{\hat{m}\times\hat{m}}, \quad \text{where} \quad \boldsymbol{\Omega}_{ij} = \boldsymbol{C}_{i-j}, \tag{5}$$

the regression coefficient matrices by

$$\Lambda_{\hat{m}} = [\boldsymbol{A}_1, \boldsymbol{A}_2, \cdots, \boldsymbol{A}_{\hat{m}}]^T, \tag{6}$$

and the right hand side of Eqs. (4) by

$$\boldsymbol{B}_{\hat{m}} = -[\boldsymbol{C}_1^T, \boldsymbol{C}_2^T, \cdots, \boldsymbol{C}_{\hat{m}}^T]^T. \tag{7}$$

Equations (4) then becomes $\boldsymbol{\Omega}_{\hat{m}\times\hat{m}}\Lambda_{\hat{m}} = \boldsymbol{B}_{\hat{m}}$. Note that (i) Eqs. (4) contain a total number of $\hat{m}N^2$ unknown regression coefficients in $\Lambda_{\hat{m}}$ and we have exactly the same number of linear equations; (ii) the coefficient matrix $\boldsymbol{\Omega}_{\hat{m}\times\hat{m}}$ in linear equations (4), in general, should be positive definite when the data length L is infinite.

Construction of positive definite matrix $\boldsymbol{\Omega}_{\hat{m}\times\hat{m}}$

As is well known, the properties of the coefficient matrix, e.g., symmetric and positive definiteness, play an important role in determining the robustness of numerical solutions to linear equations [4, 5]. Mathematically, the coefficient matrix $\boldsymbol{\Omega}_{\hat{m}\times\hat{m}}$ in Eqs. (4) is positive definite when the length of time series L is infinite. In numerical computations, however, if we first compute each covariance matrix function $\boldsymbol{C}_k$, $k = 0, 1, \cdots, \hat{m}-1$, and then construct the coefficient matrix $\boldsymbol{\Omega}_{\hat{m}\times\hat{m}}$ as in Eqs. (4) and (5), due to statistical effects caused by finite data length, $\boldsymbol{\Omega}_{\hat{m}\times\hat{m}}$ is not guaranteed to be positive definite. Therefore, when solving linear equations (4) to obtain the regression coefficients $\Lambda_{\hat{m}}$, one may obtain an incorrect inference of the causal connectivities between neurons.

To numerically construct $\boldsymbol{\Omega}_{\hat{m}\times\hat{m}}$ that preserves the property of positive definiteness, we define $\boldsymbol{Y}_t = [\boldsymbol{X}_{t+\hat{m}-1}^T, \boldsymbol{X}_{t+\hat{m}-2}^T, \cdots, \boldsymbol{X}_t^T]^T$, which is an $\hat{m}N$ dimensional vector. The coefficient matrix $\boldsymbol{\Omega}_{\hat{m}\times\hat{m}}$ can be obtained by

$$\boldsymbol{\Omega}_{\hat{m}\times\hat{m}} = \langle \boldsymbol{Y}_t \boldsymbol{Y}_t^T \rangle = \frac{1}{L-\hat{m}+1} \sum_{t=1}^{L-\hat{m}+1} \boldsymbol{Y}_t \boldsymbol{Y}_t^T. \tag{8}$$

In our cases, numerical simulations have shown that the coefficient matrix $\boldsymbol{\Omega}_{\hat{m}\times\hat{m}}$ as constructed in Eqs. (8) is always positive definite when the length of time series L is finite ($L > \hat{m}N$). After this, Eqs. (4) can be solved by many robust numerical algorithms such as the conjugate gradient method, Cholesky decomposition method or successive relaxation method [4, 5, 6].

Regression order

To obtain a good estimate of GC, it is important to determine a correct regression order m in linear regression models [7]. Some criteria have been proposed based on the information theory [8, 9]. One is the Akaike Information Criterion (AIC), which is defined by minimizing the AIC function with respect to $\hat{m}$. The AIC function is defined as

$$AIC(\hat{m}) = \ln[\det(\mathbf{\Sigma}_{\hat{m}})] + \frac{2\hat{m}N^2}{L}, \tag{9}$$

where $\det(\mathbf{\Sigma}_{\hat{m}})$ is the determinant of the covariance matrix $\mathbf{\Sigma}_{\hat{m}}$ as defined in Eqs. (2). The other criterion is the Bayesian Information Criterion (BIC) and the regression order $\hat{m}$ is chosen by minimizing the BIC function as

$$BIC(\hat{m}) = \ln[\det(\mathbf{\Sigma}_{\hat{m}})] + \frac{\hat{m}N^2 \ln L}{L}. \tag{10}$$

It has been shown that the AIC function sometimes cannot achieve a minimum when the total number of data points L is large. However, the BIC criterion can compensate for the large number of data points and usually performs better in neural applications [1]. In our study, both AIC and BIC criteria have been tested to determine the regression order $\hat{m}$, and $\hat{m}$ determined by the AIC criterion is usually larger than that obtained by using the BIC criterion. However, the final results about the inference of causal interactions between time series remain the same for both criteria.

Statistical significance

In numerical computations, due to finite data length L and finite regression order m, we can only obtain an estimate of GC, denoted by $\hat{F}$. Therefore, we should perform significance tests to determine whether the calculated nonzero $\hat{F}$ is statistically significant or it arises from statistical error. Under the null hypothesis that there is no causal influence from x_2 to x_1, the conventional large-sample distribution theory can be used [10, 2, 3] to show that $\hat{F}_{x_2\to x_1}$ is asymptotically χ^2 distributed as $L\hat{F}_{x_2\to x_1} \sim \chi^2(m)$. We can obtain a threshold for GC magnitude from x_2 to x_1 (similarly for the GC $\hat{F}_{x_1\to x_2}$) through a p-value test [11, 2, 3]. In most of our cases, p is chosen to be 0.001 in numerical simulations.

In addition, if we want to obtain the confidence interval for the theoretically defined but unknown GC, denoted by F [11, 12], The statistical significance can be similarly analyzed by using the χ^2 statistic, which is asymptotically monotonically related to the F-statistic [11, 13]. For ease of discussion, we consider the significance test for two time series $x_1(t)$ and $x_2(t)$. However, this test can be straightforwardly extended to the multivariate case.

Suppose $F_{x_1\to x_2}$ and $F_{x_2\to x_1}$ are the theoretical GC, whereas $\hat{F}_{x_1\to x_2}$ and

$\hat{F}_{x_2\to x_1}$ are the estimate of GC from empirical time series, we have

$$\begin{aligned} L\hat{F}_{x_1\to x_2} &\sim \chi'^2(m, LF_{x_1\to x_2}), \\ L\hat{F}_{x_2\to x_1} &\sim \chi'^2(m, LF_{x_2\to x_1}), \end{aligned} \tag{11}$$

where $\chi'^2(n,\lambda)$ is a noncentral χ^2 distribution with mean λ and the degree of freedom n. In practice, a direct numerical evaluation of the noncentral χ^2 distribution is usually difficult. However, there are some good approximations [14, 15]. For instance, if $x \sim \chi'^2(n,\lambda)$, then

$$\sqrt{x-(n-1)/3} \sim N(\sqrt{\lambda+(2n+1)/3}, 1) \quad \text{for} \quad x \geq (n-1)/3.$$

Using these approximations, one can obtain an approximate 95 percent confidence interval for $F_{x_1\to x_2}$ and $F_{x_2\to x_1}$ as

$$\begin{aligned} \phi_l(\hat{F}_{x_1\to x_2}, m, L) < &F_{x_1\to x_2} < \phi_r(\hat{F}_{x_1\to x_2}, m, L), \\ \phi_l(\hat{F}_{x_2\to x_1}, m, L) < &F_{x_2\to x_1} < \phi_r(\hat{F}_{x_2\to x_1}, m, L), \end{aligned}$$

where the functions $\phi_l(\cdot)$ and $\phi_r(\cdot)$ are defined as

$$\begin{aligned} \phi_l(x,m,L) &= (\sqrt{x-\frac{m-1}{3L}} - \frac{1.96}{\sqrt{L}})^2 - \frac{2m+1}{3L}, \\ \phi_r(x,m,L) &= (\sqrt{x-\frac{m-1}{3L}} + \frac{1.96}{\sqrt{L}})^2 - \frac{2m+1}{3L}. \end{aligned}$$

Minimal data length for GC reconstruction

Here, we discuss the minimal data length that is needed for a successful GC reconstruction. As an illustration of this issue, we study the two-neuron network as shown in Fig. 1A. From the conventional large-sample distribution theory [2, 3, 10], the numerical computed GC values $\hat{F}_{x_1\to x_2}$ and $\hat{F}_{x_2\to x_1}$ are asymptotically χ^2 distributed as shown in Eq. (11) and we record their probability distribution function as $\rho_1(x)$ (with causal influence) and $\rho_0(x)$ (without causal influence), respectively. As discussed in the main text, we can determine a GC threshold F_{T} by performing the p-value test. Therefore, the probability of correct topology reconstruction for this network is

$$P[\text{correct}] = \int_0^{\mathrm{F_T}} \rho_0(\mathrm{x})\mathrm{dx} \int_{\mathrm{F_T}}^{+\infty} \rho_1(\mathrm{x})\mathrm{dx} \tag{12}$$

Note that, the distributions $\rho_1(x)$ and $\rho_0(x)$ depend on the theoretical GC values $F_{x_1\to x_2}$ and $F_{x_2\to x_1}$, respectively. As discussed above, $F_{x_1\to x_2}$ and $F_{x_2\to x_1}$ are theoretically defined using infinite length of time series. Therefore, we compute GC values by using sufficiently long time series (~ 200 mins) until our numerical results are convergent and can well approximate those theoretical GC values. For a given level of correctness $P[\text{correct}]$ (e.g., 90%), Eq. (12) is an implicit function of time length [See Eq. (11)] and we can solve it using an iterative method to obtain the minimal data length $L_{\min}$ for the GC reconstruction as shown in Fig. S8.

References

[1] Ding M, Chen Y, Bressler SL (2006) Granger causality: basic theory and application to neuroscience. In Handbook of time series analysis, Schelter S, Winterhalder M, Timmer J (eds) Wiley-VCH: Berlin : 437-460.

[2] Pandit S, Wu S (1983) Time series and system analysis with applications. New York: Wiley.

[3] McQuarrie A, Tai CL (1998) Regression and time series model selection. New Jersey: World Scientific.

[4] Meyer CD (2001) Matrix Analysis and Applied Linear Algebra. Society for Industrial and Applied Mathematics.

[5] Leon SJ (2006) Linear Algebra With Applications (7th ed.). Pearson Prentice Hall.

[6] Whaley RC, Petitet A, Dongarra JJ (2001) Automated empirical optimizations of software and the atlas project. Parallel Computing 27: 3-35.

[7] Schwartz G (1978) Estimating the dimension of a model. The Annals of Statistics 5: 461-464.

[8] Akaike H (1969) Fitting autoregressive models for prediction. Annals of the Institute of Statitical Mathematics 21: 243-247.

[9] Akaike H (1978) A bayesian analysis of the minimum aic procedure. Annals of the Institute of Statitical Mathematics 30: 9-14.

[10] Greene WH (2002) Econometric analysis, fifthe ed. New Jersy: Prentice-Hall.

[11] Geweke J (1982) Measurement of linear dependence and feedback between multiple time series. Journal of the American Statistical Association 77: 304-313.

[12] Geweke J (1984) Measures of conditional linear dependence and feedback between time series. Journal of the American Statistical Association 79: 907-915.

[13] Bressler SL, Seth AK (2011) Wiener-granger causality: a well established methodology. NeuroImage 58: 323-329.

[14] Sankaran M (1963) Approximations to the non-central chi-square distribution. Biometrika 50: 199-204.

[15] Johnson NL, Kotz S (1970) Distributions in statistics: continuous univariate distributions (Vol. 2). New York: John Wiley.
